# Cancer cell‐selective ectopic expression of CD20 as an antigen enables rituximab repurposing for solid tumour immunotherapy

**DOI:** 10.1002/ctm2.70804

**Published:** 2026-08-31

**Authors:** Ziyan Kong, Yile Wang, Yunqi Zhao, Lu Wang, Zhimin Fan, Yongqian Shu, Jinke Wang

**Affiliations:** ^1^ School of Biological Science and Medical Engineering Southeast University Nanjing China; ^2^ Jiangsu Clinical Innovation Center for Anorectal Diseases of T.C.M Nanjing Hospital of Chinese Medicine Affiliated to Nanjing University of Chinese Medicine Nanjing China; ^3^ Department of Oncology The First Affiliated Hospital of Nanjing Medical University Nanjing China

**Keywords:** antibody, antigen, cancer, immunotherapy, repurpose

## Abstract

**Background:**

Despite the clinical success of cancer immunotherapies, their efficacy is often compromised by antigen‐related problems, including downregulation, loss, and off‐tumour toxicity.

**Methods:**

To overcome these limitations that challenge the current immunotherapies dependent on native antigens, we here describe a new cancer immunotherapy strategy that artificially and specifically expresses a clinically validated antigen on variant tumours and thus repurposes clinical antibody drugs to treat cancers not belonging to their indications. To authenticate the strategy, we delivered a CD20 gene under the control of an NF‐κB‐specific promoter to tumours by adeno‐associated virus and then treated them with a CD20 antibody, rituximab.

**Results:**

We found that CD20 was selectively expressed in tumours, and subsequent rituximab treatment engaged multiple antibody‐dependent effector mechanisms, including NK‐cell‐mediated ADCC and macrophage‐mediated ADCP. We demonstrated that this strategy is effective not only in variant‐cultivated cancer cells, HCT116 spheroids, and patient‐derived organoids of human colorectal cancer, but also in a humanized mouse with an HCT116 xenograft and immunocompetent mouse with an MC38 transplant. The strategy showed high cancer cell specificity in both in vitro and in vivo models, resulting in high therapeutic efficacy.

**Conclusion:**

This strategy thus creates a new modality of cancer immune‐redirection therapy by repurposing both the clinically validated antigen and antibody.

**Key points:**

- TRAP enables tumour‐selective ectopic expression of the clinically validated antigen CD20, overcoming antigen limitations in solid tumour immunotherapy.
- TRAP repurposes anti‐CD20 antibodies to engage diverse immune effector mechanisms, including ADCC, ADCP, and CDC.
- TRAP demonstrates antitumour efficacy across cellular, 3D tumour spheroid, patient‐derived organoid, and in vivo solid tumour models.

## Graphical abstract

- A recombinant AAV delivered tumour gene therapy.
- NF‐κB drives tumour‐selective antigen (e.g., CD20) expression.
- Ectopic CD20 enables rituximab repurposing to solid tumours.
- ADCC, ADCP, and CDC contribute to the antitumour activity.

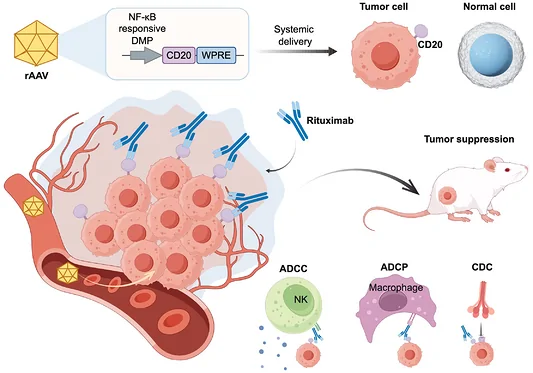

## 1 BACKGROUND

Although immunotherapy has achieved marked success in hematologic malignancies, effective antigen targeting in solid tumours remains a major challenge.^1^ In contrast to hematologic cancers, where antigens are often uniformly and specifically expressed, the heterogeneity of solid tumours poses a major barrier to discovering common therapeutic targets.^2^ Furthermore, selective pressure from targeted therapies often drives antigen downregulation or loss, leading to therapeutic resistance and posttreatment relapse.^3^, ^4^, ^5^ To overcome the antigen bottleneck, recent strategies have introduced artificial antigens into tumour cells—such as GFP via tumour‐colonizing probiotics, FITC through membrane‐inserting ligands, or VHH delivered by lipid nanoparticles—to enable immune recognition.^6^, ^7^, ^8^ Collectively, these efforts highlight the urgent need to alleviate the bottleneck in antigen selection and enable immune recognition by CAR‐T cells and therapeutic antibodies, even in the absence of native targetable antigens. Nevertheless, the clinical translation of these approaches is hindered by biosafety concerns—such as endotoxin contamination derived from Gram‐negative bacterial components and nonspecific membrane insertion of anchoring ligands—as well as by the limited stability and rapid metabolism of mRNA delivered via lipid nanoparticles.^7^, ^9^, ^10^

Antibody‐recruiting strategies have also been developed to redirect pre‐existing or vaccine‐induced humoral immunity towards tumour cells, including bifunctional viral peptide recruiters, nanobody–viral antigen chimaeras, and vaccine–antibody recruiter combinations.^11^, ^12^, ^13^ These studies highlight the potential of leveraging established humoral immunity for cancer immunotherapy. A complementary approach is to redirect antibody responses through tumour‐selective delivery of a defined target antigen. In this context, adeno‐associated virus (AAV) has emerged as a leading gene therapy platform due to its efficiency, versatility, safety, and ability to support stable transgene expression.^14^ Its clinical potential is evidenced by over 225 clinical trials and the FDA approval of multiple AAV‐based therapies, including six gene therapy products.^15^, ^16^, ^17^ Moreover, AAV‐mediated delivery has demonstrated therapeutic efficacy across a broad spectrum of diseases, from monogenic disorders to cancer.^18^, ^19^ These advantages position AAV as a robust and clinically translatable system for targeted tumour antigen delivery in cancer immunotherapy.

We previously developed an NF‐κB–responsive decoy minimal promoter (DMP), which integrates an NF‐κB decoy element with a minimal promoter to achieve cancer cell–selective transgene expression driven by aberrant NF‐κB activation.^20^, ^21^, ^22^, ^23^ Building on this system, we constructed a recombinant AAV vector in which DMP drives the expression of CD20, a clinically validated immunotherapy antigen expressed on mature B lymphocytes but absent from hematopoietic stem cells, plasma cells, and most normal tissues. CD20 has been successfully targeted by rituximab, one of the earliest approved anticancer monoclonal antibodies, and therefore serves as an ideal proof‐of‐concept antigen for evaluating tumour‐selective antigen delivery. Based on this rationale, we developed tumour‐selective antigen delivery for repurposing antibody therapy (named TRAP in concise). While CD20 and rituximab serve as a model system, TRAP can be readily adapted to other validated or engineered antigens and paired with diverse antibody modalities such as bispecifics and antibody–drug conjugates. In this way, TRAP expands the therapeutic utility of existing antibody therapies for solid tumours, while providing a flexible framework that may also be adapted for next‐generation immunotherapies.

## 2 METHODS AND MATERIALS

### 2.1 DNA constructs

The decoy minimal promoter (DMP) is an 84‐bp synthetic NF‐κB‐responsive promoter previously developed by our group.^7^ It comprises a 52‐bp NF‐κB response element containing five tandem κB‐binding sites, followed by a 32‐bp minimal promoter derived from the pGL4.10‐MP construct used in the original development of DMP. The NF‐κB response element occupies nucleotides 1–52, with the five κB‐binding sites located at nucleotides 1–10, 11–21, 22–31, 32–42, and 43–52, respectively, whereas the minimal promoter occupies nucleotides 53–84. The NF‐κB response element sequence is 5′‐GGG AAT TTC CGG GGA CTT TCC GGG AAT TTC CGG GGA CTT TCC GGG AAT TTC C‐3′, and the minimal promoter sequence is 5′‐TAG AGG GTA TAT AAT GGA AGC TCG ACT TCC AG‐3′. These sequences were used to replace the CMV promoter in the pAAV‐MCS vector (Stratagene), thereby generating the pAAV‐DMP vector. The murine CD20 coding sequence was chemically synthesized and ligated into the pAAV‐DMP plasmid using EcoRI and BamHI, resulting in the construction of pAAV‐DMP‐mCD20. To enhance transgene expression, the WPRE sequence was inserted downstream of the CD20 gene. This was achieved through homologous recombination using the ClonExpress Ultra One Step Cloning Kit (Vazyme), generating the vector pAAV‐DMP‐mCD20‐WPRE, which is also referred to as pAAV‐mCD20. Subsequently, based on the pAAV‐mCD20 plasmid, the human CD20 coding sequence was introduced by homologous recombination to generate pAAV‐DMP‐hCD20‐WPRE (pAAV‐hCD20). All primers used for plasmid construction were synthesized by Sangon Biotech (Shanghai, China) and are listed in Table S1. All plasmids, including pAAV‐MCS, pAAV‐mCD20, and pAAV‐hCD20, were transformed into E. coli DH5α (Vazyme), purified using EndoFree Plasmid Kits (Vazyme), and verified by DNA sequencing.

### 2.2 Cell culture and isolation of PBMC

The cell lines used in this study included HEK‐293T (human fetal kidney cells), HCT116 (human colon cancer cells), PANC‐1 (human pancreatic cancer cells), MDA‐MB‐231 (human breast cancer cells), MC38 (mouse colon cancer cells), HL7702 (human normal hepatocytes), GES‐1 (human gastric mucosal epithelial cells), MCF‐12A (human breast epithelial cells), and NIH‐3T3 (mouse embryonic fibroblasts). HEK‐293T, PANC‐1, MDA‐MB‐231, MC38, HL7702, GES‐1, and NIH‐3T3 cell lines were obtained from the cell‐resource centre of Shanghai Institutes for Biological Sciences, Chinese Academy of Sciences. HCT116 and MCF‐12A cell lines were acquired from American Type Culture Collection (ATCC). HEK‐293T, HCT116, PANC‐1, MDA‐MB‐231, and NIH‐3T3 cells were cultured in Dulbecco's Modified Eagle Medium (DMEM; Gibco), and HL7702, GES‐1, MCF‐12A, and MC38 cells were cultured in Roswell Park Memorial Institute (RPMI) 1640 medium (Gibco). All media were supplemented with 10% fetal bovine serum, 100 U/mL penicillin, and 100 µg/mL streptomycin (Gibco). Cells were incubated at 37°C in a humidified incubator containing 5% CO_2_. Whole blood from healthy donors was processed by density‐gradient centrifugation using SepMate tubes and Lymphoprep (STEMCELL Technologies). After centrifugation, the PBMC layer was collected, washed with RPMI 1640 containing 5 mM EDTA and 2% FBS, and resuspended in RPMI 1640 supplemented with 10% FBS.

### 2.3 Cell transfection

Cell transfection was performed using Lipofectamine 2000 (Thermo Fisher Scientific, USA) according to the manufacturer's instructions. Briefly, 5 × 10^5^ cells were seeded into six‐well plates and incubated overnight. The next day, cells were transfected separately with 4 µg of pAAV‐MCS, pAAV‐mCD20, or pAAV‐hCD20 plasmids. After transfection, cells were cultured for an additional 72 h before being harvested for flow cytometry. All samples were resuspended in flow cytometry staining buffer (FACS buffer), stained with anti‐human CD20‐APC (clone 2H7, BioLegend, USA) or anti‐mouse CD20‐PE (clone QA18A73, BioLegend) for 30 min at 4°C in the dark, washed three times, and then resuspended in FACS buffer before analysis.

### 2.4 CD107a expression and IFN‐γ staining

CD107a and IFN‐γ expression were commonly used as indicators of NK cell functional and cytotoxic activity.^24^, ^25^ Therefore, CD107a degranulation and IFN‐γ production assays were performed using PBMCs that had been pre‐activated overnight with 50 U/mL IL‐2 (R&D Systems) and served as effector cells.

Tumour and normal cells transfected with either pAAV‐MCS or pAAV‐hCD20 for 72 h were used as target cells. These cells were first incubated with rituximab (10 µg/mL) at 37°C for 30 min. PBMCs were then harvested, washed with culture medium, and co‐cultured with the target cells at an effector‐to‐target (E:T) ratio of 30:1. To assess degranulation, anti‐CD107a‐FITC antibody (clone H4A3, BioLegend) and 1× Monensin Solution (BioLegend) were added at the beginning of the co‐culture. After 4 h of incubation at 37°C in a humidified 5% CO_2_ incubator, cells were collected and first stained with anti‐CD3‐PE (clone SK7, BioLegend) and anti‐CD56‐APC antibodies (clone 5.1H11, BioLegend) to identify NK cell populations. Cells were then fixed and permeabilized using the Cyto‐Fast Fix/Perm Buffer Set (BioLegend), followed by intracellular staining with anti‐IFN‐γ‐FITC antibody (clone 4S.B3, BioLegend) (1:20 dilution) at 4°C for 30 min. After washing, samples were analyzed by flow cytometry using a NovoCyte 2000R instrument (Agilent Technologies).

### 2.5 In vitro ADCC assays

ADCC was measured using a lactate dehydrogenase (LDH) release assay. Tumour cells and normal cells transfected with plasmids (pAAV‐MCS or pAAV‐hCD20) were used as target cells. After 72 h of transfection, the cells were incubated with rituximab (10 µg/mL) at 37°C for 30 min, followed by co‐culture with PBMCs pre‐activated overnight with 50 U/mL IL‐2 (R&D Systems) at an effector‐to‐target (E:T) ratio of 30:1. After 4 h of incubation at 37°C, cell‐free supernatants were collected to assess target cell lysis by measuring LDH release using the CytoTox 96 Non‐Radioactive Cytotoxicity Assay (Promega), according to the manufacturer's instructions. The percentage of cytotoxicity was calculated using the following formula: Cytotoxicity (%) = (sample LDH release—spontaneous LDH release from effector and target cell co‐culture)/(maximum LDH release from target cells—spontaneous LDH release from target cells) × 100. All experiments were performed in triplicate.

### 2.6 Apoptosis assay

HCT116 cells transfected with pAAV‐MCS or pAAV‐hCD20 for 72 h were treated with or without rituximab (10 µg/mL) for 4 h in the absence of PBMCs. Cells were stained using an Annexin V‐FITC/PI Apoptosis Detection Kit (KeyGEN BioTECH, Jiangsu, China) according to the manufacturer's instructions and analyzed by flow cytometry. Apoptosis was defined as the percentage of Annexin V^+^ cells (Annexin V^+^PI^−^ and Annexin V^+^PI^+^).

### 2.7 ADCP assay

Antibody‐dependent cellular phagocytosis (ADCP) was evaluated using a flow cytometry‐based assay adapted from Hong et al.^26^ THP‐1 cells were differentiated into macrophage‐like cells by treatment with PMA (50 ng/mL) for 24 h. HCT116 cells were transfected with pAAV‐MCS or pAAV‐hCD20 for 72 h and then labelled with DiO. PMA‐differentiated THP‐1 macrophage‐like cells were labelled with CellTrace Far Red and co‐cultured with DiO‐labelled HCT116 cells at an effector‐to‐target ratio of 2:1 in the presence or absence of rituximab (10 µg/mL). After 6 h of co‐culture, cells were collected and analyzed by flow cytometry. ADCP was calculated as: ADCP (%) = Q2 / (Q1 + Q2) × 100, where Q2 represents DiO^+^ CellTrace Far Red^+^ double‐positive events, and Q1 represents DiO^+^ CellTrace Far Red^−^ target‐cell events.

### 2.8 C3 fragment deposition and CDC assays

Tumour cells transfected with plasmids encoding either control vector (pAAV‐MCS) or human CD20 (pAAV‐hCD20) were used 72 h post‐transfection. Ofatumumab was used in the complement assays because of its stronger CDC‐inducing capacity than rituximab, thereby providing a sensitive functional readout of TRAP‐induced CD20 expression.^27^, ^28^ For evaluation of complement activation, transfected tumour cells were pre‐incubated with ofatumumab (10 µg/mL) for 15 min at 37°C in 5% CO_2_, followed by the addition of normal human serum (NHS) or heat‐inactivated human serum (IHS) as a complement source to a final concentration of 25% and further incubation for 30 min at 37°C. Cells were then harvested, washed with PBS containing 2% BSA, and stained for deposited C3 fragments (clone 3E7, anti‐C3b/iC3b; BioLegend). Cell‐surface C3 fragment deposition was quantified by flow cytometry.

CDC was further assessed by measuring LDH release. Transfected tumour cells were pre‐incubated with ofatumumab for 15 min at 37°C, followed by the addition of NHS or IHS and incubation for 3 h at 37°C. LDH released into the supernatant was quantified using the CytoTox 96® Non‐Radioactive Cytotoxicity Assay (Promega) according to the manufacturer's instructions.

### 2.9 Virus preparation

Recombinant adeno‐associated virus serotype 2 (rAAV2) vectors were generated using a helper‐free packaging system. HEK293T cells were seeded into 75 cm^2^ flasks at a density of 5 × 10^6^ cells per flask and cultivated overnight. Cells were then co‐transfected with pHelper, pAAV‐RC, and one of the pAAV transfer plasmids (pAAV‐MCS, pAAV‐hCD20 or pAAV‐mCD20) using ExFect Transfection Reagent (Vazyme) according to the manufacturer's instructions. The pAAV‐RC plasmid provided the AAV2‐derived *rep* and *cap* genes for rAAV2 packaging. After 72 h of incubation, cells and culture medium were harvested and frozen at −80°C overnight. The frozen material was subsequently thawed in a 37°C water bath to promote cell lysis, and the freeze–thaw cycle was repeated three times. Following lysis, pure chloroform (1/10 of the total volume) was added to the lysate, and the mixture was incubated at 37°C for 1 h with vigorous shaking. Sodium chloride was then added to the solution to a final concentration of 1 M, and the mixture was gently mixed until fully dissolved. It was centrifuged at 12 000×*g* for 15 min at 4°C, and the supernatant was collected. Polyethylene glycol 8000 (PEG8000) was added to the supernatant to a final concentration of 10% (w/v) and stirred until completely dissolved. After centrifugation (12 000×*g*, 15 min, 4°C), the resulting pellet was resuspended in PBS. To remove residual nucleic acids, DNase and RNase were added to a final concentration of 1 µg/mL and incubated at room temperature for 30 min. The solution was then extracted once with an equal volume of chloroform, and the aqueous phase containing the purified virus was transferred to a new tube. AAV titres were determined by quantitative PCR using AAV‐F and AAV‐R primers (Table S2). Virus aliquots were stored at −80°C until use. The resulting viruses were designated rAAV‐MCS, rAAV‐hCD20, and rAAV‐mCD20.

### 2.10 Experiments in tumour spheroids

HCT116 cells were cultured as tumour spheroids using a serum‐free suspension method. Briefly, cells were resuspended at a density of 1 × 10^4^ cells/mL in serum‐free DMEM/F12 medium (Gibco) supplemented with 20 ng/mL epidermal growth factor (EGF, PeproTech), 20 ng/mL basic fibroblast growth factor (bFGF, PeproTech), 1× B27 supplement (Gibco), 1× N2 supplement (Gibco), and 1% penicillin–streptomycin (Gibco). A volume of 100 µL of the cell suspension was dispensed into each well of an ultra‐low attachment 96‐well plate (Corning, USA) and incubated at 37°C in a humidified incubator with 5% CO_2_. Tumour spheroids were allowed to form for approximately 48 h. Spheroid formation was monitored by microscopy, and uniform, compact spheroids were selected for subsequent experiments.

Western blot analysis was employed to detect target antigen expression in tumour cells. Established spheroids were infected with either rAAV‐MCS or rAAV‐hCD20 at a dose of 1 × 10^8^ viral genomes (vg) per well. At 72 h post‐infection, spheroids were harvested to evaluate CD20 protein expression by Western blotting, thereby assessing the ability of rAAV to penetrate and transduce tumour spheroids. Total protein was extracted using a Total Protein Extraction Kit (Solarbio, China) following the manufacturer's instructions. Equal amounts of protein (20 µg per sample) were resolved by SDS‐PAGE and transferred to membranes for immunoblotting. Membranes were probed with a mouse monoclonal anti‐β‐actin antibody (Wuhan Sanying, China) (1:10 000) and a monoclonal anti‐CD20 antibody (Wuhan Sanying) (1:5000). IRDye 800CW goat anti‐mouse IgG (C80118‐05, LI‐COR; 1:10 000) was used as the secondary antibody. Protein bands were visualized and quantified using the Odyssey Infrared Imaging System (LI‐COR) and Odyssey software.

Cytotoxicity assays in HCT116 spheroids stained using a Calcein/PI Cell Viability/Cytotoxicity Assay Kit (Beyotime, China). The spheroids were infected with either rAAV‐MCS or rAAV‐hCD20 at a dose of 1 × 10^8^ viral genomes (vg) per well and cultured for an additional 72 h. Following viral transduction, spheroids were treated with 10 µg/mL rituximab (MCE, USA) at 37°C for 30 min, and then co‐cultured with 5 × 10^4^ PBMCs (pre‐activated overnight with 50 U/mL IL‐2; R&D Systems) per well for 24 h. This corresponded to an E:T ratio of 50:1, calculated based on the initial seeding of 1×10^3^ HCT116 cells per well. Following 24 h of PBMC co‐culture, low‐magnification brightfield images covering multiple spheroids in each treatment group were acquired using a 4× objective. After co‐culture, spheroids were gently washed with PBS and stained with 100 µL of staining solution from the Calcein/PI Cell Viability/Cytotoxicity Assay Kit (Beyotime). Samples were incubated at 37°C in a humidified 5% CO_2_ incubator for 30 min. Optical and fluorescence images were acquired using a fluorescence microscope. Cell viability was quantified by measuring the fluorescence area of Calcein (live cells) and PI (dead cells) using ImageJ software (NIH, USA). Projected spheroid area and Calcein AM/PI fluorescence signals were quantified using ImageJ, and measurements from evaluable spheroids within each of three independent experiments were averaged to generate one independent data point.

### 2.11 3D organoid culture

PDOs of colorectal cancer were established from surgical resection specimens of colorectal cancer patients at the Nanjing Hospital of Traditional Chinese Medicine, according to previously described protocols.^29^ Organoids were embedded in organoid culture ECM (reduced growth factor; bioGenous, China) and maintained in colorectal cancer organoid medium (serum‐free; bioGenous) at 37°C in a humidified incubator with 5% CO_2_. The medium was refreshed every 2–3 days, and organoids were passaged approximately every 7 days by mechanical dissociation.

### 2.12 Assessment of antibody‐dependent cytotoxicity against organoids

Organoids were dissociated into single cells using TrypLE Express (Gibco, USA) and seeded at a density of 1 × 10^5^ cells per well in ultra‐low‐attachment 24‐well plates (Corning, USA), followed by incubation with 500 µL of organoid culture medium overnight. RAAV‐MCS and rAAV‐hCD20 were then added to each well at a dose of 1 × 10^10^ vg per well, together with 500 µL of fresh organoid culture medium, and the cultures were maintained for an additional 72 h. On the day of the cytotoxicity assay, organoids were collected and labelled with 5 µM CellTracker Red CMTPX dye (Thermo Fisher Scientific) in organoid culture medium without growth factors at 37°C for 30 min. After washing, organoids were pre‐incubated with rituximab diluted in colorectal cancer organoid medium to a final concentration of 10 µg/mL for 30 min at 37°C. Approximately 20 organoids per well were then seeded into ultra‐low‐attachment 96‐well plates. Organoid culture ECM was added to reach a final concentration of 10%. Caspase‐3/7 activity was selected as a mechanistically relevant readout of apoptosis induced by antibody‐dependent immune cytotoxicity in intact organoids, consistent with previous organoid‐based NK‐cell and ADCC studies.^30^ To quantify apoptosis, Caspase‐3/7 Green dye (Thermo Fisher Scientific) was added to each well at a final concentration of 5 µM. PBMCs and Caspase‐3/7 Green dye were added immediately, and the co‐culture plates were centrifuged at 300×*g* for 3 min to ensure that NK cells and organoids were localized in the same focal plane. Each organoid was estimated to contain approximately 50 cells. Accordingly, co‐culturing 20 organoids with 5 × 10^4^ PBMCs corresponded to an approximate effector‐to‐target (E:T) ratio of 50:1. After 24 h of co‐culture, fluorescence images were acquired using a fluorescence microscope. Regions of interest (ROIs) were defined based on the CellTracker Red signal to outline individual organoids, and the mean fluorescence intensity of the Caspase‐3/7 signal within each ROI was quantified using ImageJ software to assess treatment‐induced apoptosis.

### 2.13 Quantitative PCR

Total RNA was extracted from cell lines and mouse tissues using TRIzol reagent (Invitrogen) according to the manufacturer's instructions. Complementary DNA (cDNA) was generated using the PrimeScript RT Reagent Kit with gDNA Eraser (Takara). Genomic DNA (gDNA) was isolated from mouse tissues using the TIANamp Genomic DNA Kit (TIANGEN). Quantitative PCR (qPCR) was performed on an ABI StepOne Plus system (Applied Biosystems) using Fast SYBR Green Master Mix (Roche). Each sample was analyzed in triplicate, and all experiments were independently repeated at least three times. Relative mRNA expression levels were calculated using the 2^−ΔC^ *^t^* or 2^−ΔΔC^ *^t^* method, where ΔC*t* = C*t*_target_ − C*t*_GAPDH_ and ΔΔC*t* = ΔC*t*_treatment_ − ΔC*t*_control_. The 2^−ΔΔC^ *^t^* value was also defined as the relative quantity (RQ). Viral vector DNA abundance was quantified using AAV‐F and AAV‐R primers designed to amplify a fragment within the human growth hormone (hGH) polyadenylation signal of the packaged rAAV vector genome. Viral DNA abundance was normalized to GAPDH as the internal control and expressed as RQ = 2^−ΔC^ *^t^*. Primer specificity was confirmed by melting curve analysis, and the sequences of all primers are provided in Table S2.

### 2.14 Single‐cell RNA‐sequencing analysis

Publicly available single‐cell RNA‐sequencing data from human pancreatic ductal adenocarcinoma (GSE335452) were analyzed to compare RELA expression between tumour and non‐tumour cells. Cell distributions were visualized by t‐SNE, and RELA expression was displayed using a dot plot.

### 2.15 Animal models

Ten‐week‐old female C57BL/6J mice were purchased from Changzhou Cavens Laboratory Animal Co., Ltd. (China). Xenograft mouse models were generated using six‐week‐old female NOD CRISPR Prkdc Il2r gamma (NCG) triple‐immunodeficient mice obtained from GemPharmatech Co., Ltd. (China). All mice were housed in a specific pathogen‐free (SPF) animal facility. All animal experiments were conducted in accordance with the guidelines and ethical standards of the Animal Care and Use Committee of Southeast University (Nanjing, China). Tumour growth was monitored by calliper measurements, and tumour volumes were calculated using the formula *V* = (*ab* ^2^)/2, where *a* represents the longest diameter and *b* the shortest diameter. Mice were euthanized when tumour volume reached 2000 mm^3^ or when body weight loss exceeded 20% of baseline weight. Various tissues, including the heart, liver, spleen, lung, kidney, and tumour, were collected for subsequent analyses.

Humanized mouse models were performed in six‐week‐old female NCG mice bearing subcutaneous hind‐flank tumours established from HCT116 colorectal cancer cells. Mice were randomly assigned into three groups (*n* = 6). Group 1 received 5 × 10^6^ HCT116 cells in 100 µL PBS. Groups 2 and 3 received 5 × 10^6^ HCT116 cells mixed with 1 × 10^6^ PBMCs in 100 µL PBS. When tumours reached an average volume of ∼100 mm^3^, Groups 2 and 3 were intravenously injected with either rAAV‐MCS or rAAV‐hCD20 (5 × 10^10^ vg/mouse). rAAV was administered every other day for a total of two injections. Two days after the final rAAV injection, Groups 2 and 3 were treated with rituximab (30 mg/kg). Tumour size and body weight were measured every 2 days. Mice were euthanized when tumour volume reached 2000 mm^3^. Various tissues, including the heart, liver, spleen, lung, kidney, and tumour, were harvested for viral DNA and gene expression analyses. The heart, liver, spleen, lung, and kidney were processed for H&E staining, whereas tumour tissues were subjected to TUNEL staining. Serum samples from each group were collected for biochemical assays.

For syngeneic models, MC38 cells were washed with PBS, resuspended at 1 × 10^7^ cells/mL, and subcutaneously implanted into the inguinal region of 10‐week‐old female C57BL/6J mice (1 × 10^6^ cells in 100 µL per mouse). Animals were randomly assigned to five treatment groups (rAAV‐MCS, rAAV‐mCD20, anti‐mouse CD20 mAb, rAAV‐MCS + anti‐mouse CD20 mAb, and rAAV‐mCD20 + anti‐mouse CD20 mAb; *n* = 6 per group). Treatment was initiated when tumour volumes reached ∼100 mm^3^. Mice received intravenous injections of 100 µL rAAV‐MCS or rAAV‐mCD20 (5 × 10^10^ vg/mouse) every day for a total of two doses, followed 48 h later by intravenous administration of anti‐mouse CD20 mAb (MB20‐11, 250 µg/mouse in 100 µL PBS) via the lateral tail vein. Tumour size and body weight were measured daily. Mice were euthanized when tumour volume reached 2000 mm^3^. Various tissues, including the heart, liver, spleen, lung, kidney, and tumour, were harvested for H&E staining, viral DNA quantification, and gene expression analyses. Blood and serum samples were collected from each group for routine haematological tests and serum biochemical assays. To further evaluate the effect of this therapy on mouse survival, Kaplan‐Meier analysis was performed. MC38 tumour‐bearing mice were randomly assigned to three treatment groups (PBS, rAAV‐MCS + anti‐mouse CD20 mAb, and rAAV‐mCD20 + anti‐mouse CD20 mAb; *n* = 6 per group). Mice received intravenous injections of rAAV‐MCS or rAAV‐mCD20 (5 × 10^10^ vg/mouse) every day for a total of two doses, followed 48 h later by intravenous administration of anti‐mouse CD20 mAb (250 µg/mouse) via the lateral tail vein. Subsequent antibody doses were administered once weekly for two additional injections. Mice were euthanized when tumour volume reached 2000 mm^3^, and survival was recorded accordingly.

To evaluate the contribution of FcγR‐dependent effector mechanisms, a separate FcγR blockade experiment was performed in MC38 tumour‐bearing mice. Mice were randomly assigned to three groups (*n* = 6 per group): rAAV‐MCS + mCD20 Ab, rAAV‐mCD20 + mCD20 Ab, and rAAV‐mCD20 + mCD20 Ab + FcγR blockade. rAAV and mCD20 Ab were administered as described above. For FcγR blockade, mice received an FcγR‐blocking antibody (clone 9E9, 150 µg/mouse, i.v.) 24 h before mCD20 Ab administration. Tumour growth was monitored, and tumour weight and volume were measured at the experimental endpoint.

To further evaluate TRAP efficacy in a microsatellite‐stable (MSS) colorectal cancer model, a syngeneic CT26 tumour model was established in 6‐week‐old female BALB/c mice. CT26 cells (1 × 10^6^ cells/mouse) were subcutaneously implanted, and mice were randomly assigned into two groups (*n* = 6 per group). When tumour volumes reached approximately 100 mm^3^, mice received an intravenous injection of rAAV‐MCS or rAAV‐mCD20 (5 × 10^10^ vg/mouse). Anti‐mouse CD20 mAb (MB20‐11, 250 µg/mouse) was subsequently administered intravenously on days 10 and 17 after tumour implantation. Tumour volume and body weight were monitored throughout the experiment. Mice were euthanized when tumour volume reached 2000 mm^3^, and tumours were collected for measurement of tumour volume and weight.

### 2.16 Ex vivo tumour processing and immunophenotyping

To evaluate the impact of rAAV‐hCD20 on human NK cells, HCT116 tumours were harvested on day 4 following rituximab (RTX) administration. Tumour‐infiltrating lymphocytes were isolated by mechanical dissociation using a gentle MACS Dissociator (Miltenyi Biotec), followed by filtration through 70‐µm cell strainers and washing with PBS. The resulting cell suspension was resuspended in staining buffer (BioLegend) containing human and mouse Fc receptor blocking reagents (Human TruStain FcX and anti‐mouse CD16/32, BioLegend) and stained with anti‐human CD45‐FITC (clone HI30, BioLegend), CD3‐PE (clone SK7, BioLegend), and CD56‐APC (clone 5.1H11, BioLegend). Dead cells were excluded using 7‐AAD Viability Staining Solution (BioLegend) during flow cytometric analysis. Gating was performed by first selecting live CD45^+^ cells, followed by singlet gating, and subsequent identification of NK cells as CD3^−^CD56^+^ events. NK‐cell frequency was calculated as the percentage of CD3^−^CD56^+^ cells within the single, live CD45^+^ population.

To assess DMP‐driven mCD20 expression at the tumour‐cell level in vivo, a separate short‐term experiment was performed in MC38 tumour‐bearing mice. When tumour volumes reached approximately 100 mm^3^, mice received rAAV‐MCS or rAAV‐mCD20 (5 × 10^10^ vg/mouse; *n* = 3 per group), and tumours were harvested one week after rAAV administration. Tumours were dissociated into single‐cell suspensions, and after exclusion of debris, doublets, dead cells, and CD45^+^ leukocytes, surface mCD20 expression was analyzed within viable CD45^−^ tumour cells by flow cytometry.

To characterize the tumour immune microenvironment in the immunocompetent MC38 model, tumours were collected 4 days after anti‐mouse CD20 antibody administration from mice treated with rAAV‐MCS + anti‐mouse CD20 mAb or rAAV‐mCD20 + anti‐mouse CD20 mAb (*n* = 3 per group). Tumours were dissociated into single‐cell suspensions and filtered through 70 µm cell strainers. After dead‐cell exclusion and Fc receptor blocking with anti‐mouse CD16/32 antibody, cells were stained with the indicated fluorophore‐conjugated antibodies (Table S3). T cells were defined as CD45^+^CD3^+^ cells, NK cells as CD45^+^CD3^−^NK1.1^+^ cells, and macrophages as CD45^+^CD11b^+^F4/80^+^ cells, with CD86 expression assessed within the macrophage population. Polymorphonuclear myeloid‐derived suppressor cells (PMN‐MDSCs) were defined as CD45^+^CD11b^+^Ly6G^+^Ly6C^low/int^ cells, whereas monocytic myeloid‐derived suppressor cells (M‐MDSCs) were defined as CD45^+^CD11b^+^Ly6G^−^Ly6C^high^ cells.

Livers were collected from the same mice 4 days after anti‐mouse CD20 antibody administration and dissociated into single‐cell suspensions. After excluding debris, doublets, and dead cells and Fc receptor blocking, cells were stained with anti‐mouse CD45‐FITC, CD31‐APC, and CD20‐PE antibodies. Surface mCD20 expression was analyzed in hepatic CD45^+^ and CD31^+^ cell populations by flow cytometry.

### 2.17 Histological and immunohistochemical staining

Tissues including the heart, liver, spleen, lung, and kidney were harvested, fixed, embedded in paraffin, sectioned, and stained with haematoxylin and eosin (H&E) using standard histological procedures. Briefly, dissected tissues were fixed in 4% paraformaldehyde (Sangon Biotech) at room temperature overnight, then embedded in paraffin and sectioned at a thickness of 5 µm. The sections were stained with haematoxylin solution (C0107, Beyotime) followed by eosin solution (Beyotime). For CD20 immunohistochemistry, paraffin‐embedded tumour and major‐organ sections were processed using standard procedures and stained with an anti‐CD20 primary antibody (clone SB237, Servicebio, China), followed by DAB visualization. Images were acquired using a light microscope (IX51, Olympus).

### 2.18 TUNEL assay

Terminal deoxynucleotidyl transferase dUTP nick‐end labelling (TUNEL) assay was performed to detect apoptosis in xenografted HCT116 tumour tissues according to the manufacturer's instructions (Beyotime). In brief, paraffin‐embedded sections were deparaffinized, rehydrated, and incubated with TUNEL reaction solution, followed by counterstaining with haematoxylin for 5 min. TUNEL‐positive cells were visualized under a light microscope at 100× magnification. Quantification of positively stained cells was conducted using ImageJ software (version 1.53t, NIH).

For assessment of hepatic apoptosis, liver sections were subjected to TUNEL staining together with immunofluorescence staining for CD45 (SB723, Servicebio) or CD31 (SB149, Servicebio) followed by DAPI nuclear counterstaining.

### 2.19 Flow cytometry

All antibodies used for flow cytometry are listed in Table S3. Flow cytometry data were acquired using either a NovoCyte 2000R flow cytometer (Agilent Technologies) or a CytoFLEX S flow cytometer (Beckman Coulter), as appropriate, and analyzed using FlowJo v10.8 software.

### 2.20 Statistical analysis

All data are presented as mean ± standard deviation (SD). Statistical analyses and graph generation were performed using GraphPad Prism 8.0 software. Statistical differences between two groups were evaluated using a two‐tailed Student's *t*‐test. For comparisons involving three or more groups, one‐way or two‐way analysis of variance (ANOVA) was performed, followed by Tukey's or Sidak's multiple comparisons test when appropriate. Animal survival differences were analyzed using the Kaplan–Meier method, and *p*‐values were calculated by the log‐rank (Mantel–Cox) test. *p*‐value of less than .05 was considered statistically significant.

## 3 RESULTS

### 3.1 Conceptualization of TRAP

**FIGURE 1:**
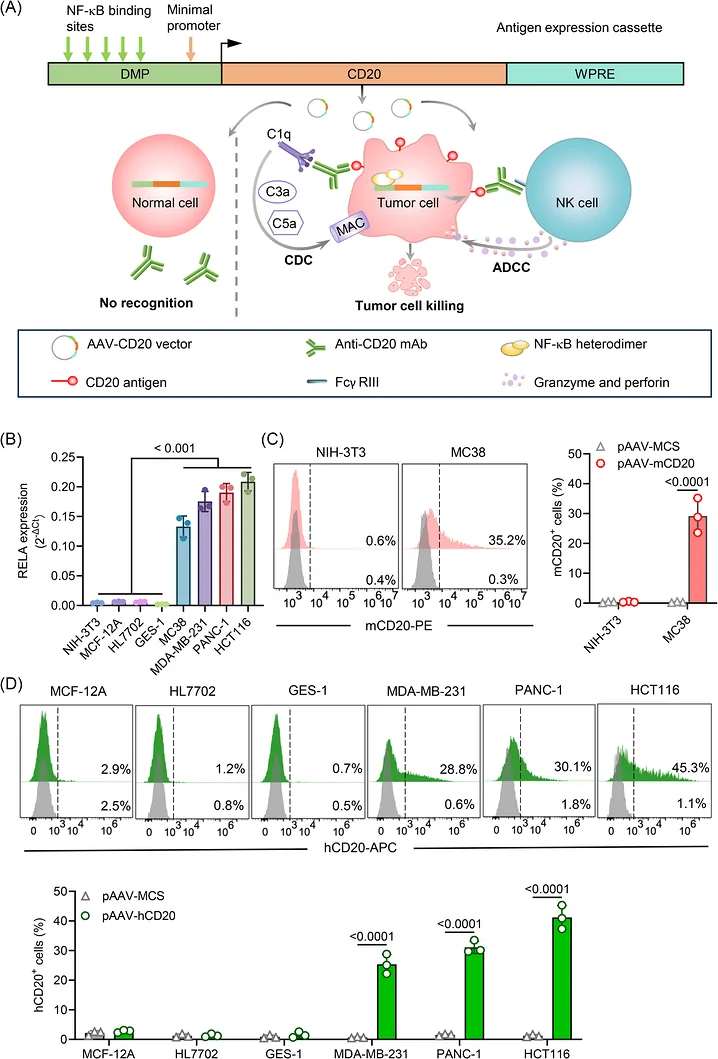
TRAP design and DMP‐driven CD20 expression in tumour versus normal cells. (A) Schematic of the TRAP system. The CD20 coding sequence was placed under the control of an NF‐κB‐responsive decoy minimal promoter (DMP) containing multiple NF‐κB binding sites, with WPRE downstream to enhance expression. In tumour cells with constitutive NF‐κB activity, DMP drives CD20 surface expression, enabling recognition by an anti‐CD20 monoclonal antibody (anti‐CD20 mAb) and Fcγ receptor III (CD16)–mediated ADCC, whereas normal cells with low NF‐κB activity remain unrecognized. In parallel, engagement of the anti‐CD20 mAb can recruit C1q via its Fc domain to activate the classical complement cascade, leading to C3b/iC3b deposition and membrane attack complex (MAC) formation, thereby inducing complement‐dependent cytotoxicity (CDC). (B) qPCR analysis of RELA (p65) mRNA in tumour (HCT116, MDA‐MB‐231, PANC‐1, MC38) and normal (MCF‐12A, HL7702, GES‐1, NIH‐3T3) cell lines. Mean ± SD, *n* = 3 independent experiments. One‐way ANOVA (two‐sided) with Tukey's test. (C) Flow‐cytometry analysis of murine CD20 (mCD20) expression in MC38 and NIH‐3T3 cells transfected with pAAV‐mCD20 or pAAV‐MCS. Representative histograms with the percentages of mCD20^+^ cells indicated (left), and quantification of mCD20^+^ cells (right) are shown. (D) Flow‐cytometry analysis of human CD20 (hCD20) expression in tumour (HCT116, MDA‐MB‐231, PANC‐1) and normal (MCF‐12A, HL7702, GES‐1) cell lines transfected with pAAV‐hCD20 or pAAV‐MCS. Representative histograms with the percentages of hCD20^+^ cells indicated (top), and quantification of hCD20^+^ cells (bottom) are shown. Data in (C) and (D) are presented as mean ± SD (*n* = 3). Statistical significance was assessed using an unpaired two‐tailed Student's *t*‐test.

The mechanism of TRAP is illustrated schematically in Figure 1A. TRAP consists of two components: an adeno‐associated virus (AAV) vector that delivers the antigen and a monoclonal antibody specific for the target antigen. In this proof‐of‐concept study, we selected CD20—a clinically validated transmembrane tumour antigen—as the model target. To generate the AAV expression vector, the CMV promoter in the pAAV‐MCS (multiple cloning site) backbone was replaced with a synthetic NF‐κB–responsive decoy minimal promoter (DMP), and the CD20 coding sequence was inserted downstream of DMP. To enhance transgene expression, the woodchuck hepatitis virus posttranscriptional regulatory element (WPRE) was placed immediately downstream of the CD20 gene and upstream of the poly(A) signal. This configuration yielded the plasmid pAAV‐DMP‐CD20‐WPRE, which was subsequently used for AAV packaging (AAV‐CD20). The DMP contains five tandem NF‐κB‐binding sites followed by a minimal promoter. Mechanistically, activated NF‐κB binds to these sites and promotes transcription from the adjacent minimal promoter, thereby coupling downstream CD20 expression to intracellular NF‐κB activity. In the tumour models examined in this study, elevated NF‐κB activity therefore resulted in preferential DMP‐driven CD20 expression. As illustrated in Figure 1A, once CD20 is displayed on the tumour cell surface, an anti‐CD20 monoclonal antibody (anti‐CD20 mAb) binds to the antigen. Antibody Fc domains recruit C1q and activate the classical complement cascade, resulting in cleavage of C3 and subsequent activation of the terminal complement pathway, with generation of the anaphylatoxins C3a and C5a, as well as deposition of C3b/iC3b fragments on the tumour cell surface. This process can proceed to formation of the membrane attack complex (MAC) and ultimately lead to complement‐dependent cytotoxicity (CDC). In parallel, the Fc portion of antibody‐bound CD20 is recognized by Fcγ receptor III (FcγRIII, CD16) on natural killer (NK) cells, thereby triggering NK‐cell activation and polarized degranulation, leading to the release of perforin and granzymes and culminating in antibody‐dependent cellular cytotoxicity (ADCC). In contrast, normal cells lacking CD20 expression are not recognized and remain unaffected.

### 3.2 Targeted surface expression of CD20 in tumour cells by the DMP promoter in vitro

To evaluate whether NF‐κB activity can be exploited for tumour‐selective transgene expression, we first quantified RELA/p65 expression across representative tumour and normal cell lines. QPCR analysis revealed that RELA was consistently higher in tumour cell lines (HCT116, MDA‐MB‐231, PANC‐1, and MC38) compared with normal counterparts (MCF‐12A, HL7702, GES‐1, and NIH‐3T3) (Figure 1B). Analysis of the independent single‐cell RNA‐sequencing dataset GSE335452 further showed higher RELA expression in tumour cells than in non‐tumour cells (Figure S1). To test whether this difference in NF‐κB activity could drive CD20 expression, we transfected cells with AAV plasmids carrying either the human CD20 (pAAV‐hCD20) or murine CD20 (pAAV‐mCD20) coding sequence under the control of the DMP promoter, depending on species origin. Flow cytometry analysis demonstrated marked induction of CD20‐positive cells in tumour cell lines, whereas only low background positivity was observed in normal cell lines (Figure 1C,D). For instance, MC38 cells transfected with pAAV‐mCD20 exhibited strong mCD20 induction, whereas NIH‐3T3 cells remained negative (Figure 1C). Similarly, DMP‐driven hCD20 was abundantly expressed in HCT116, MDA‐MB‐231, and PANC‐1 cells, but absent in MCF‐12A, HL7702, and GES‐1 cells (Figure 1D). By contrast, cells transfected with the empty pAAV‐MCS vector showed no detectable CD20, confirming that expression required the presence of the DMP‐CD20 construct. These findings show that DMP activation relies on hyperactive NF‐κB in tumour cells, leading to CD20 expression in malignant but not normal cells.

### 3.3 Antitumour effect of TRAP in vitro

**FIGURE 2:**
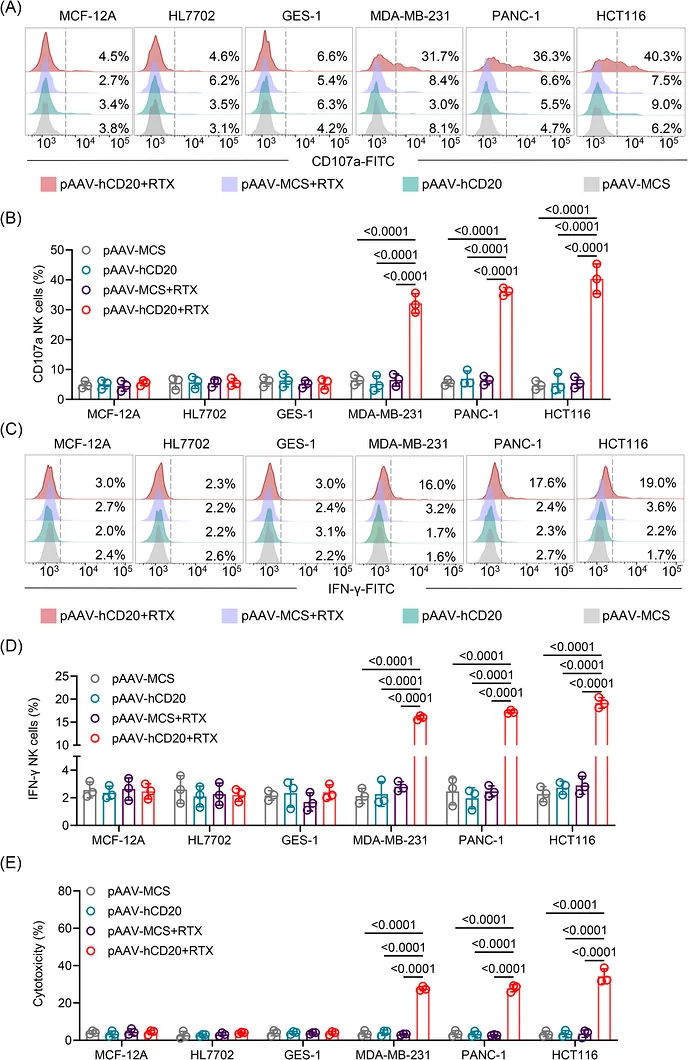
NK‐cell degranulation, IFN‐γ production and ADCC with CD20‐expressing target cells. (A) Representative flow‐cytometry histograms of CD107a expression in NK cells (CD3^−^CD56^+^; gating in Figure S2) after co‐culture with normal (MCF‐12A, HL7702, GES‐1) and tumour (MDA‐MB‐231, PANC‐1, HCT116) cells transfected with pAAV‐MCS or pAAV‐hCD20, with or without rituximab (RTX). Percentages of CD107a^+^ NK cells are indicated. (B) Quantification of CD107a^+^ NK cells in (A). Mean ± SD, *n* = 3 independent experiments. One‐way ANOVA (two‐sided) with Tukey's test within each cell line. (C) Representative histograms of intracellular IFN‐γ expression in NK cells under the same conditions as in (A). (D) Quantification of IFN‐γ^+^ NK cells in (C). Mean ± SD, *n* = 3. Statistical analysis as in (B). **(E)** ADCC assessed by LDH release after co‐culture of IL‐2‐activated PBMCs with CD20‐expressing or control cells in the presence or absence of rituximab (RTX). Mean ± SD, *n* = 3. Statistical analysis as in (B). PBMCs were pre‐activated overnight with IL‐2 and co‐cultured with targets at an effector‐to‐target (E:T) ratio of 30:1 for 4 h after RTX pre‐incubation.

Having confirmed selective CD20 expression in tumour cells, we next investigated whether this expression could be functionally exploited by rituximab (RTX) to activate NK cells. Tumour and normal cell lines transfected with pAAV‐hCD20 or vector control were co‐cultured with IL‐2‐activated PBMCs in the presence or absence of RTX. NK‐cell activity was assessed by CD107a surface mobilization, indicative of degranulation, and intracellular IFN‐γ production, reflecting effector cytokine release. NK cells were identified as CD3^−^CD56^+^ lymphocytes according to the gating strategy shown in Figure S2. In tumour cell lines (MDA‐MB‐231, PANC‐1, HCT116), NK cells exhibited significantly elevated CD107a and IFN‐γ expression only when pAAV‐hCD20 transfection was combined with RTX, whereas neither CD20 expression alone nor RTX treatment alone produced comparable activation (Figure 2A–D). These findings demonstrate that both the introduced target and the therapeutic antibody are required for NK‐cell engagement. By contrast, three normal cell lines (MCF‐12A, HL7702, GES‐1) showed no significant changes in CD107a or IFN‐γ under any condition, consistent with their low NF‐κB activity and absence of DMP‐driven CD20 expression.

LDH‐release assays further showed enhanced antibody‐dependent cytotoxicity under TRAP conditions. Robust ADCC was observed in tumour cells only when CD20 expression and RTX were combined, whereas either component alone induced minimal lysis (Figure 2E). In normal cell lines, no significant cytotoxicity was detected under any treatment condition. Together, these results establish that TRAP translates selective CD20 expression into functional immune recognition. By requiring both antigen expression and RTX engagement, TRAP enables potent NK‐cell activation and antibody‐dependent cytotoxicity in tumour cells while sparing normal counterparts. To assess direct rituximab‐induced apoptosis, pAAV‐MCS‐ or pAAV‐hCD20‐transfected HCT116 cells were treated with rituximab in the absence of PBMCs. Annexin V/PI analysis showed no significant increase in apoptosis in pAAV‐hCD20‐transfected cells after rituximab treatment (Figure S3A,B), indicating that direct rituximab‐induced apoptosis was not detectable under these conditions.

**FIGURE 3:**
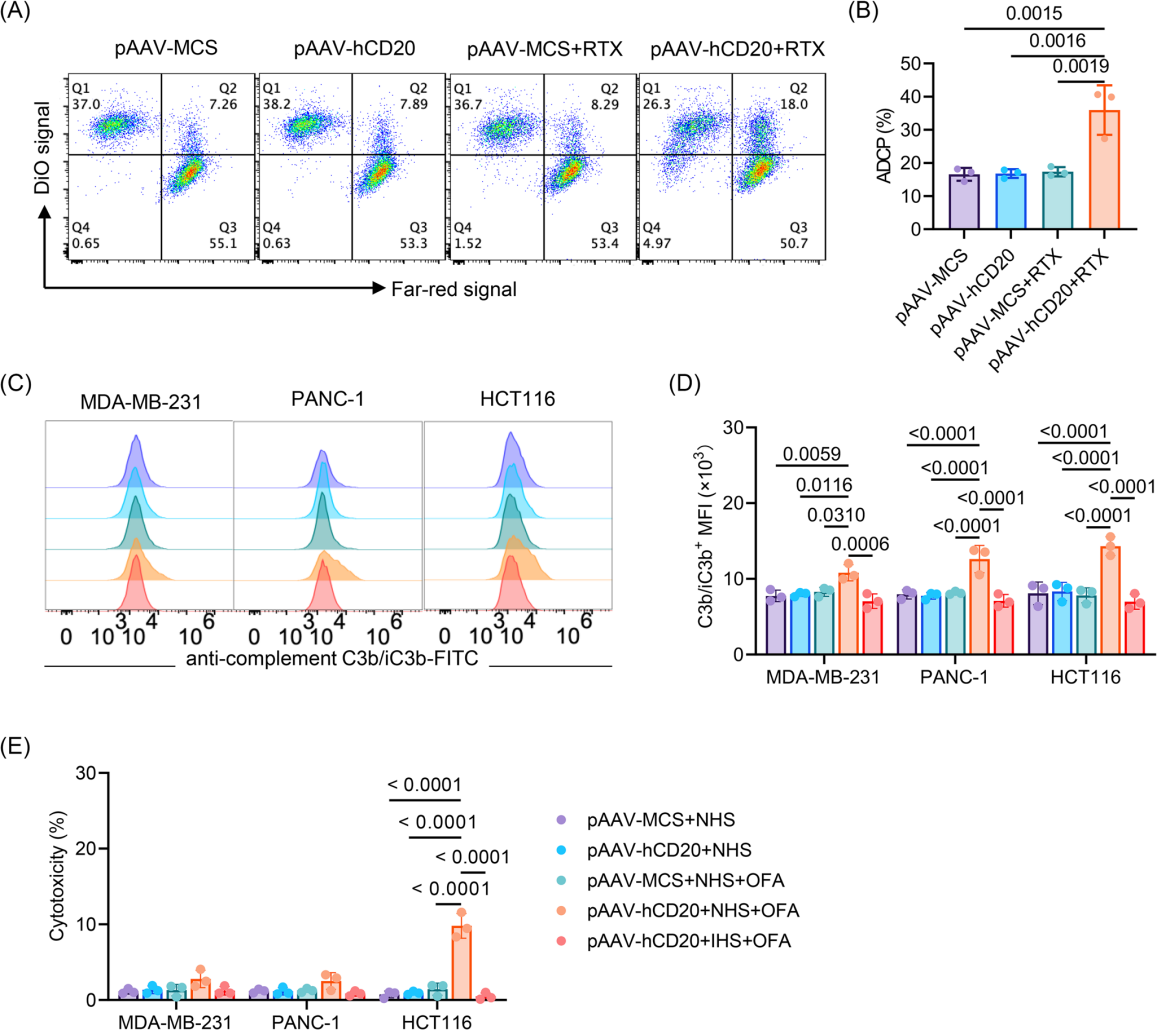
TRAP‐mediated ADCP and complement activation in tumour cells. (A) Representative flow cytometry plots of antibody‐dependent cellular phagocytosis (ADCP). DiO‐labeled HCT116 cells transfected with pAAV‐MCS or pAAV‐hCD20 were co‐cultured with CellTrace Far Red‐labelled PMA‐differentiated THP‐1 macrophage‐like cells in the presence or absence of rituximab (RTX). (B) Quantification of ADCP under the indicated conditions. Data are presented as mean ± SD, *n* = 3 independent experiments. One‐way ANOVA (two‐sided) with Tukey's test. (C) Representative flow cytometry histograms showing cell‐surface deposition of complement C3 fragments (C3b/iC3b) on MDA‐MB‐231, PANC‐1, and HCT116 cells transfected with pAAV‐hCD20 or vector control (pAAV‐MCS). Cells were treated with ofatumumab (OFA) in the presence of normal human serum (NHS) or heat‐inactivated human serum (IHS) as indicated and stained with an anti‐C3b/iC3b antibody. (D) Flow cytometric quantification of cell‐surface deposition of complement C3 fragments (C3b/iC3b) on MDA‐MB‐231, PANC‐1, and HCT116 cells transfected with pAAV‐MCS or pAAV‐hCD20 following treatment with OFA in the presence of NHS or IHS, as indicated. Data are shown as MFI. Mean ± SD, *n* = 3 independent experiments. One‐way ANOVA (two‐sided) with Tukey's test within each cell line. (E) Complement‐dependent cytotoxicity (CDC) assessed by LDH release in MDA‐MB‐231, PANC‐1, and HCT116 cells transfected with pAAV‐MCS or pAAV‐hCD20 following OFA treatment in the presence of NHS or IHS, as indicated. Data are presented as cytotoxicity (%). Mean ± SD, *n* = 3 independent experiments. Statistical analysis as in (D).

In addition to NK cell‐mediated ADCC, we further evaluated whether TRAP could induce macrophage‐mediated ADCP. DiO‐labelled HCT116 cells transfected with pAAV‐MCS or pAAV‐hCD20 were co‐cultured with CellTrace Far Red‐labelled PMA‐differentiated THP‐1 macrophage‐like cells in the presence or absence of rituximab. Flow cytometry showed that the pAAV‐hCD20 + THP‐1 + rituximab group exhibited a markedly higher ADCP level than the control groups (Figure 3A,B). These results indicate that TRAP‐mediated CD20 expression enables rituximab to trigger macrophage‐mediated phagocytosis of tumour cells.

In parallel with ADCC and ADCP, we examined whether TRAP‐mediated CD20 expression could also engage and activate the complement system. Complement activation was first evaluated by flow cytometric analysis of cell‐surface deposition of C3 fragments (C3b/iC3b) on pAAV‐hCD20‐transfected tumour cells (MDA‐MB‐231, PANC‐1, and HCT116) following treatment with an anti‐CD20 monoclonal antibody (ofatumumab, OFA) in the presence of normal human serum (NHS). TRAP‐mediated CD20 expression combined with OFA treatment in the presence of NHS resulted in a significant increase in C3b/iC3b deposition. In contrast, pAAV‐MCS‐transfected tumour cells did not show a significant increase in C3 fragment deposition following OFA treatment in the presence of NHS, with C3b/iC3b levels remaining at baseline levels observed in the pAAV‐MCS + NHS, pAAV‐hCD20 + NHS, and pAAV‐hCD20 + heat‐inactivated human serum (IHS) + OFA groups (Figure 3C,D).

We next assessed whether complement activation translated into tumour cell lysis by measuring LDH release. Despite an increase in C3 fragment deposition following OFA treatment in the presence of NHS, CDC was observed only in CD20‐expressing HCT116 cells, whereas MDA‐MB‐231 and PANC‐1 cells did not exhibit a significant increase in LDH release under the same conditions (Figure 3E). To further verify the complement dependence of the observed cytotoxicity, NHS was replaced with IHS. Under these conditions, CDC was no longer significant, indicating that the cytotoxic effect observed in HCT116 cells required an intact complement system.

### 3.4 TRAP‐mediated cytotoxicity in 3D tumour spheroid and patient‐derived organoid models

**FIGURE 4:**
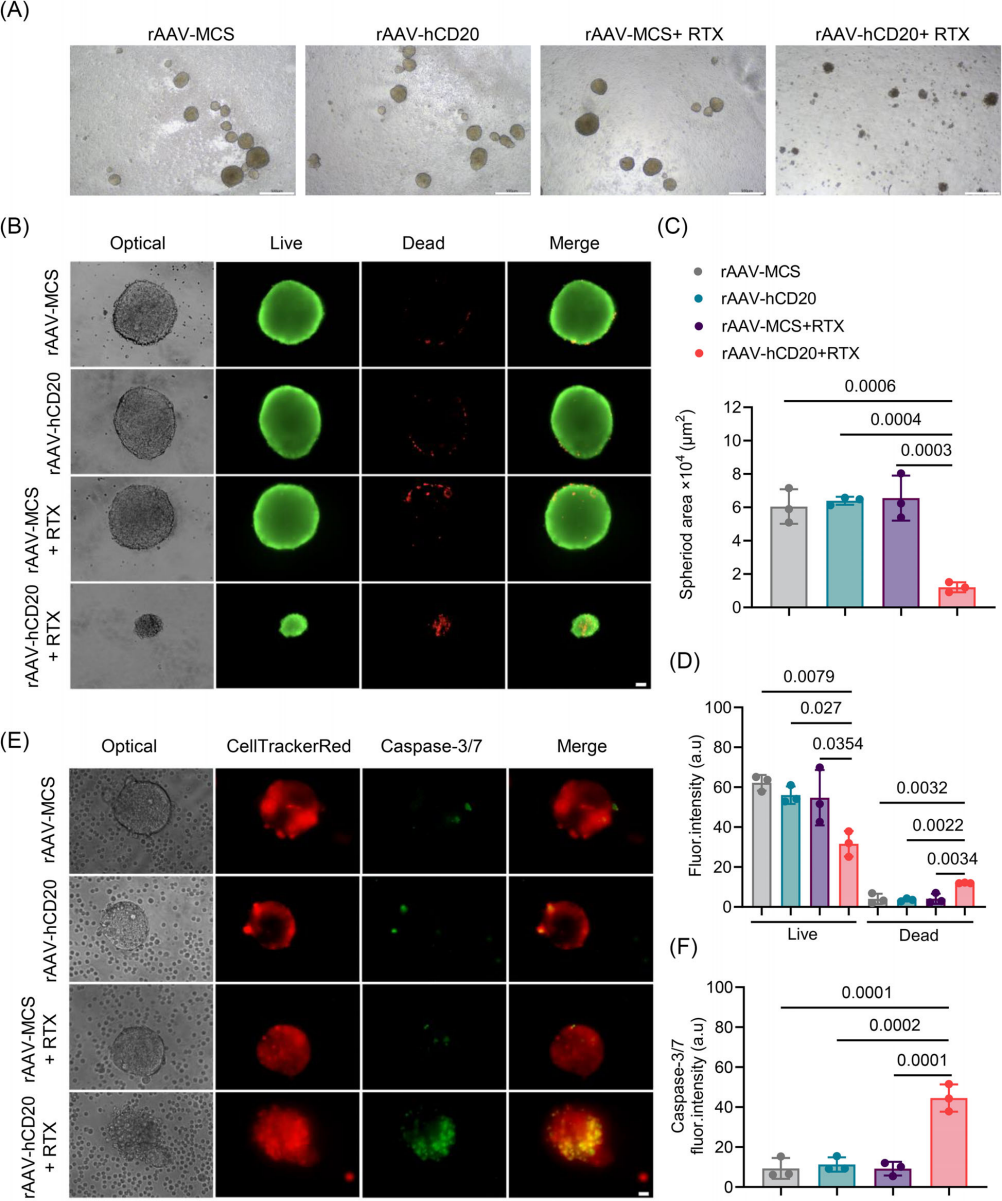
TRAP‐mediated cytotoxicity in 3D HCT116 spheroids and patient‐derived colorectal cancer organoids. **(A)** Representative low‐magnification brightfield images of HCT116 spheroids treated with rAAV‐MCS, rAAV‐hCD20, rAAV‐MCS + RTX, or rAAV‐hCD20 + RTX. Images were acquired using a 4× objective after 24 h of PBMC co‐culture and show multiple spheroids in each treatment group. Scale bar = 500 µm. (B) Representative brightfield and fluorescence images of HCT116 spheroids treated with rAAV‐MCS, rAAV‐hCD20, rAAV‐MCS + rituximab (RTX), or rAAV‐hCD20 + RTX. Live and dead cells were stained with Calcein AM (green) and propidium iodide (PI) (red), respectively. Scale bar, 50 µm. (C) Quantification of spheroid area under the indicated conditions. Bars represent mean ± SD (*n* = 3 independent experiments). Statistical significance was determined using one‐way ANOVA (two‐sided) with Tukey's multiple‐comparison test. (D) Quantification of Calcein (live) and PI (dead) fluorescence intensities in spheroids across treatment groups. Data represent mean ± SD (*n* = 3 independent experiments). Statistical analysis was performed using one‐way ANOVA (two‐sided) with Tukey's test. (E) Representative brightfield and fluorescence images of patient‐derived colorectal cancer organoids treated with rAAV‐MCS, rAAV‐hCD20, rAAV‐MCS + RTX, or rAAV‐hCD20 + RTX. Organoids were transduced with rAAV, pre‐incubated with RTX, and co‐cultured with PBMCs for 24 h. Organoids were pre‐labelled with CellTracker Red CMTPX (red), and apoptotic cells were visualized using Caspase‐3/7 Green (green). Scale bar, 20 µm. (F) Quantification of Caspase‐3/7 fluorescence intensity within organoid regions of interest (ROIs) defined by CellTracker Red. Data represent mean ± SD (*n* = 3 independent experiments). Statistical significance was assessed using one‐way ANOVA (two‐sided) with Tukey's multiple‐comparison test.

To further assess TRAP efficacy in a three‐dimensional context that better reflects solid tumour architecture, we established HCT116 spheroids under serum‐free suspension culture conditions and applied rAAV‐based gene delivery. Specifically, pAAV‐MCS and pAAV‐hCD20 plasmids were packaged into recombinant adeno‐associated virus (rAAV) vectors, designated rAAV‐MCS and rAAV‐hCD20, and subsequently used to infect established spheroids at a dose of 1 × 10^8^ viral genomes (vg) per well. Western blot analysis at 72 h post‐infection confirmed robust CD20 protein expression in rAAV‐hCD20‐treated spheroids, whereas no signal was detected in rAAV‐MCS controls, indicating that rAAV efficiently penetrates the spheroid mass and mediates gene expression (Figure S4). We next evaluated whether CD20 expression rendered spheroids susceptible to RTX‐dependent killing. Following viral transduction, spheroids were treated with rituximab (RTX, 10 µg/mL) and co‐cultured with IL‐2‐activated PBMCs (E:T ≈ 50:1) for 24 h. Low‐magnification brightfield images showed that spheroids in the rAAV‐MCS, rAAV‐hCD20, and rAAV‐MCS + RTX groups generally retained relatively intact morphology, whereas those in the rAAV‐hCD20 + RTX group exhibited marked shrinkage, structural disruption, and fragmentation (Figure 4A). Viability was assessed using Calcein AM and propidium iodide (PI) staining. As shown in Figure 4B, spheroids treated with rAAV‐MCS alone, rAAV‐hCD20 alone, or rAAV‐MCS plus RTX maintained compact morphology with strong Calcein fluorescence and minimal PI uptake. In contrast, spheroids receiving rAAV‐hCD20 together with RTX exhibited marked structural collapse, reduced Calcein signal, and increased PI staining. Quantitative analysis corroborated these observations, showing a significant decrease in spheroid area under TRAP conditions compared with all controls (Figure 4C), and fluorescence measurements revealed a pronounced reduction in live‐cell signal accompanied by elevated dead‐cell staining (Figure 4D). These findings extend the two‐dimensional results in Figure 2 to a more physiologically relevant three‐dimensional model, demonstrating that rAAV‐hCD20 delivery enables RTX‐dependent immune cytotoxicity against tumour spheroids.

To further validate TRAP efficacy in clinically relevant models, patient‐derived organoids (PDOs) of colorectal cancer established from surgical resection specimens were transduced with rAAV‐hCD20 at 1 × 10^10^ vg per well for 72 h, followed by RTX‐mediated cytotoxicity assays. Organoids were pre‐labelled with CellTracker Red CMTPX (5 µM) and pre‐incubated with RTX (10 µg/mL, 30 min) before co‐culture with PBMCs (E:T ≈ 50:1) for 24 h. Apoptosis was detected using Caspase‐3/7 Green fluorescence imaging. Similar to spheroids, organoids infected with rAAV‐hCD20 exhibited strong Caspase‐3/7 activation and structural disintegration in the presence of RTX and PBMCs, whereas those treated with rAAV‐MCS, RTX alone, or rAAV‐hCD20 alone maintained intact morphology with minimal apoptotic signal (Figure 4E). Quantification of Caspase‐3/7 fluorescence intensity within CellTracker Red‐defined regions of interest (ROIs) confirmed significantly higher apoptosis in the rAAV‐hCD20 + RTX group compared with all controls (Figure 4F). Collectively, these results demonstrate that TRAP‐mediated antigen delivery confers sensitivity of both tumour spheroids and patient‐derived colorectal cancer organoids to RTX‐dependent immune cytotoxicity, thereby providing strong support for the translational potential of TRAP in structured tumour environments.

### 3.5 Virus‐based TRAP antitumour in vivo

**FIGURE 5:**
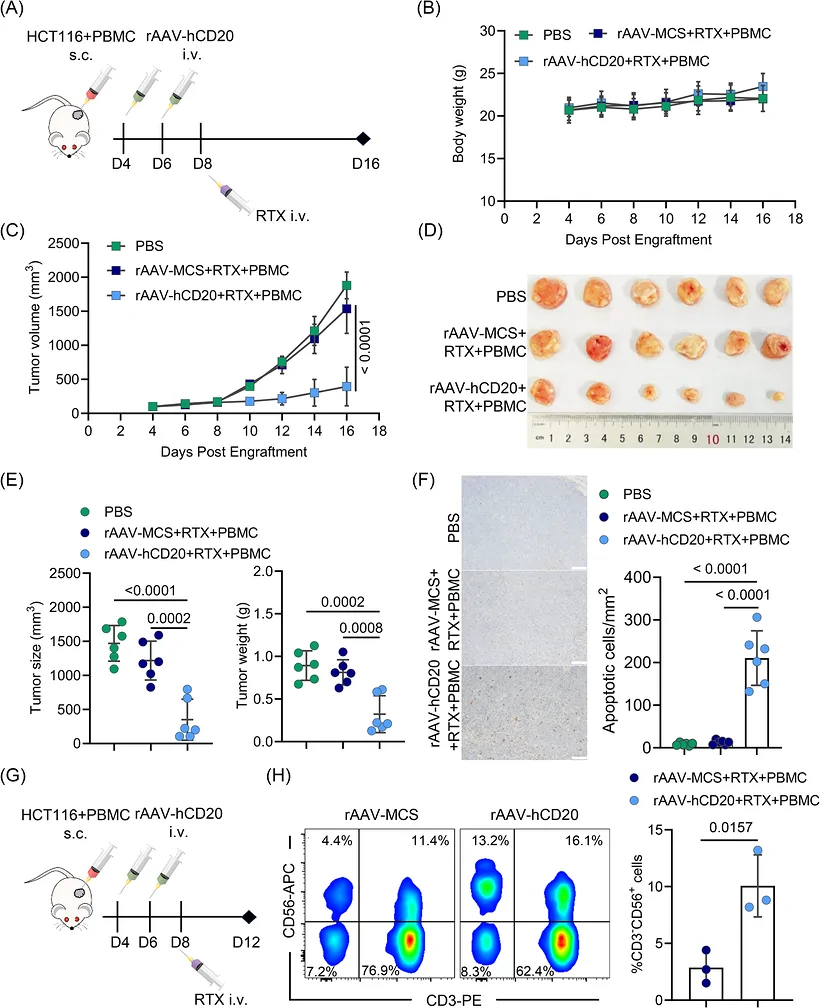
TRAP inhibits tumour growth in a humanized HCT116 xenograft model. (A) Schematic of experimental design. NCG mice were subcutaneously (s.c.) implanted with HCT116 cells and human PBMCs, followed by intravenous (i.v.) injection of rAAV‐hCD20 and rituximab (RTX). (B) Mouse body weight monitored during treatment (*n* = 6). (C) Tumour growth curves of each treatment group (*n* = 6). (D) Images of excised tumours collected from each group at endpoint. (E) Quantification of tumour size (left) and tumour weight (right) (*n* = 6). (F) TUNEL staining of tumour sections and quantification of apoptotic cells. Scale bar, 100 µm. (G) Schematic of tumour‐infiltrating lymphocyte (TIL) collection on day 4 after RTX administration. (H) Flow cytometry plots showing intratumoral NK cells, defined as CD3^−^CD56^+^ cells within the viable CD45^+^ population, and quantification of NK‐cell frequency (*n* = 3). Data are shown as mean ± SD. Statistical significance was analyzed by one‐way ANOVA with Tukey's test in (C), (E), and (F), and by the unpaired two‐tailed Student's *t*‐test in (H).

Building on the significant in vitro antitumour activity, we next assessed the in vivo therapeutic potential of the TRAP platform using a humanized HCT116 xenograft model established in NOD CRISPR Prkdc Il2rγ (NCG) mice. NCG mice were randomly assigned to three groups and subcutaneously implanted with HCT116 cells. Group 1 received tumour cells alone and served as the control group, whereas Groups 2 and 3 were co‐injected with HCT116 cells and human PBMCs as described in Section 2. When tumours reached approximately 100 mm^3^, Groups 2 and 3 were treated with rAAV‐MCS or rAAV‐hCD20, respectively, in combination with rituximab (5 × 10^10^ vg/mouse AAV; 30 mg/kg RTX) (Figure 5A). Body weight was monitored throughout the study as an indicator of general health, and no significant weight loss was observed in any group (Figure 5B). Treatment with rAAV‐MCS + rituximab (Group 2) showed no discernible effect on tumour growth compared with the control group, indicating that the presence of PBMCs alone did not influence tumour progression in this model. In contrast, rAAV‐hCD20 combined with rituximab (Group 3) resulted in a significant reduction in tumour volume, suggesting that tumour‐specific CD20 expression enabled effective redirection of rituximab, leading to potent in vivo antitumour activity (Figure 5C‒E).

To investigate the mechanism underlying this tumour inhibition, we performed terminal deoxynucleotidyl transferase dUTP nick end labelling (TUNEL) staining on tumour sections to assess treatment‐induced cell death. Group 3 exhibited a markedly higher number of TUNEL‐positive cells compared with the other groups, indicating enhanced apoptosis following TRAP treatment (Figure 5F). To assess the in vivo biodistribution of rAAV and hCD20 expression, we quantified rAAV DNA abundance and measured hCD20 mRNA expression in tumours and major organs, including the heart, lung, kidney, spleen, and liver. As a result, rAAV DNA was detected in all examined tissues (Figure S5A). However, hCD20 mRNA expression was substantially elevated only in tumour tissue (Figure S5B). Consistent with these results, immunohistochemical staining confirmed hCD20 protein expression in rAAV‐hCD20‐treated tumours, whereas no appreciable specific hCD20 staining was detected in the examined major organs. The intratumoral hCD20 staining showed a nonuniform spatial distribution (Figure S5C).

We next examined intratumoral NK‐cell frequency 4 days after rituximab administration to determine the impact of TRAP treatment on NK‐cell persistence (Figure 5G‒H; Figure S6). NK‐cell frequency was significantly higher in the rAAV‐hCD20 + rituximab group compared with the rAAV‐MCS + rituximab group (Figure 5H), suggesting that TRAP may support NK‐cell retention within the tumour microenvironment. Together with the increased apoptosis observed in Group 3, these findings indicate a possible involvement of NK cells in mediating TRAP‐associated antitumour activity. Regarding biosafety, histological analysis revealed no pathological abnormalities in the heart, liver, spleen, lung, or kidney (Figure S7A). Additionally, TRAP treatment did not cause significant changes in liver function markers (ALT, AST, ALP) or kidney function indicators (BUN, CRE, UA) compared with the PBS control group (Figure S7B).

**FIGURE 6:**
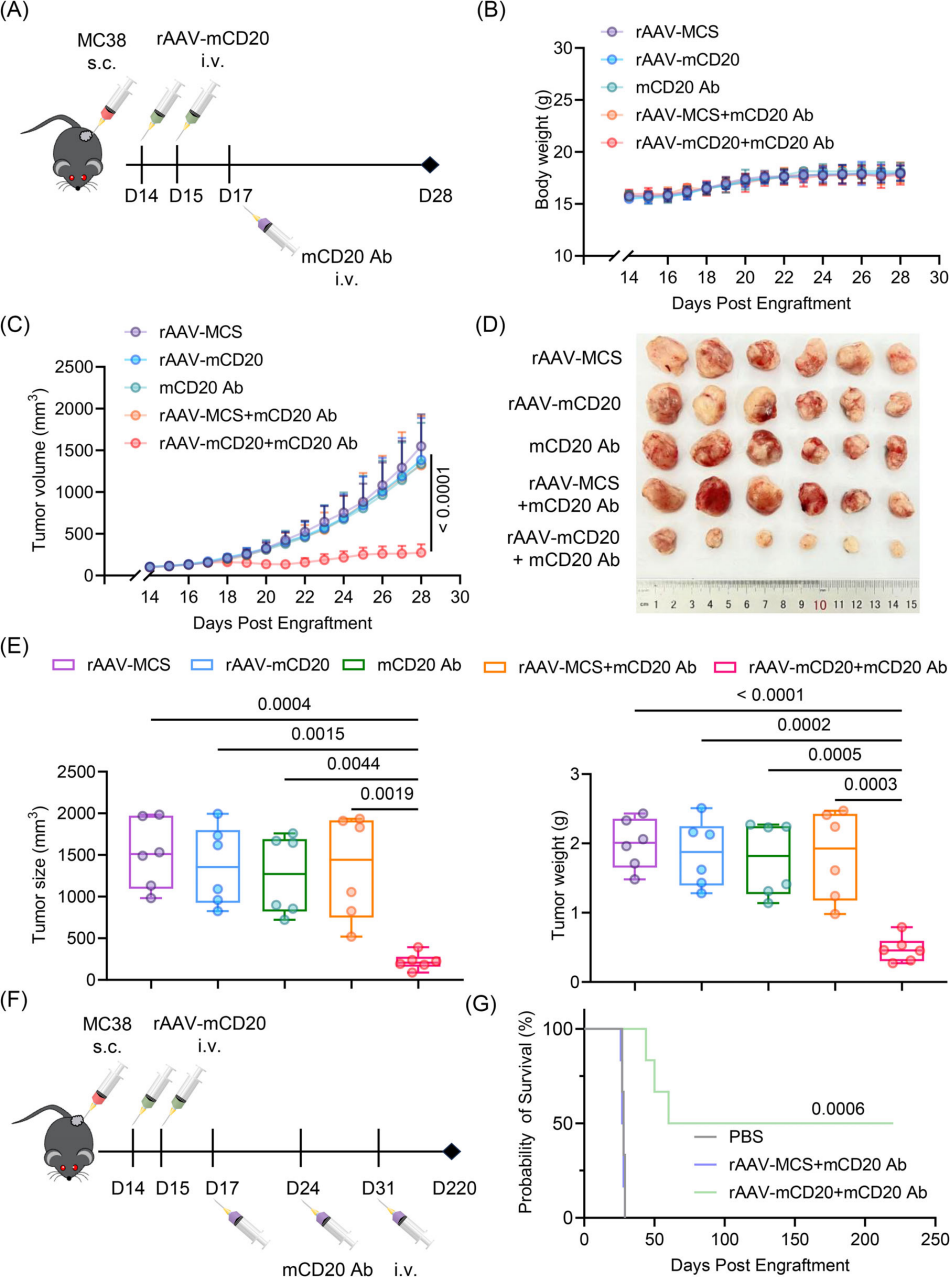
TRAP suppresses tumour progression in an immunocompetent MC38 model. (A) Schematic of experimental design. C57BL/6 mice were subcutaneously (s.c.) implanted with MC38 cells and received intravenous (i.v.) administration of AAV vectors and anti‐mouse CD20 antibody (mCD20 Ab) as indicated. (B) Mouse body weight during treatment (*n* = 6). (C) Tumour volume curves for rAAV‐MCS, rAAV‐mCD20, mCD20 Ab, rAAV‐MCS + mCD20 Ab, and rAAV‐mCD20 + mCD20 Ab (*n* = 6). (D) Images of excised tumours from each group at endpoint. (E) Tumour size (left) and tumour weight (right) at endpoint (*n* = 6). (F, G) Treatment schedule and survival analysis. **(F)** Schematics of animal treatment. **(G)** Kaplan–Meier survival curve (*n* = 6). Data are shown as mean ± SD. Statistical significance was analyzed by one‐way ANOVA with Tukey's test in (C) and (E), and by the log‐rank test in (G).

Although immunocompromised mice facilitate the evaluation of human PBMC responses in the rAAV platform, we next aimed to assess the TRAP system within an immunocompetent host bearing an intact tumour microenvironment. To this end, the pDMP‐mCD20 plasmid was cloned into an AAV backbone to generate recombinant vectors (rAAV‐mCD20), enabling in vivo validation of TRAP‐mediated antitumour efficacy. A subcutaneous colon cancer model was established by injecting MC38 cells into C57BL/6 mice (Figure 6A). Animals were randomly assigned to five treatment groups (rAAV‐MCS, rAAV‐mCD20, anti‐mouse CD20 monoclonal antibody (mCD20 Ab), rAAV‐MCS + mCD20 Ab, and rAAV‐mCD20 + mCD20 Ab; *n*  =  6 per group), as described in Section 2. Throughout the treatment period, mouse body weight remained stable across all groups (Figure 6B), indicating no overt toxicity. Notably, the combination of rAAV‐mCD20 and mCD20 Ab significantly inhibited tumour growth and led to a marked reduction in final tumour weight (Figure 6C‒E). In contrast, neither rAAV‐mCD20 nor mCD20 Ab monotherapy exhibited any measurable antitumour effect. These results underscore the necessity of both tumour‐specific antigen expression and antibody administration to achieve therapeutic efficacy. To further characterize the treatment effects, we analyzed the distribution of rAAV DNA and the expression of mRNA for RELA and the target gene *mCD20* across multiple tissues, including the heart, liver, spleen, lung, kidney, and tumour. rAAV DNA was detected in all examined tissues, with particularly high levels observed in the liver and tumour (Figure S8A). *RELA* mRNA was significantly more abundant in tumour tissue than in other organs, consistent with the known overactivity of NF‐κB in tumours (Figure S8B). In the groups not receiving mCD20 Ab, relatively high splenic mCD20 expression primarily reflected endogenous CD20‐expressing B cells. In contrast, the reduced splenic mCD20 expression in all mCD20 Ab‐treated groups was consistent with antibody‐mediated depletion of these cells (Figure S8C). Immunohistochemical staining further showed a heterogeneous spatial distribution of mCD20 within rAAV‐mCD20‐treated MC38 tumours (Figure S8D). In a separate short‐term experiment, tumours were harvested one week after rAAV administration, and flow cytometric analysis showed that approximately 40% of viable CD45^−^ tumour cells exhibited detectable surface mCD20 expression following rAAV‐mCD20 administration, compared with background‐level staining in the rAAV‐MCS group (Figure S8E,F). These findings indicate heterogeneous DMP‐driven mCD20 expression within MC38 tumours in vivo. To further assess potential off‐target mCD20 expression in the liver, we analyzed hepatic CD45^+^ immune and CD31^+^ cell populations. Flow cytometric analysis showed no significant increase in surface mCD20 expression in either population in the rAAV‐mCD20 + mCD20 Ab group compared with the rAAV‐MCS + mCD20 Ab group (Figure S9A–C). Combined immunofluorescence and TUNEL staining likewise showed no obvious increase in apoptotic signals associated with CD45^+^ or CD31^+^ cells in the liver (Figure S9D,E). To further evaluate the safety profile of the TRAP regimen, we analyzed a comprehensive panel of haematological and biochemical parameters in treated mice, including red blood cell count (RBC), platelet count (PLT), haemoglobin (HGB), liver function indicators (ALT, AST, and ALP), and kidney function markers (BUN, CRE, UA). Except for a reduction in white blood cell counts, no significant abnormalities were observed (Figure S10A). This decrease was anticipated, as CD20‐directed therapy eliminates B cells, a clinically recognized and reversible effect, with compensatory recovery expected over time. Histopathological examination of major organs (heart, liver, lung, kidney) revealed no pathological changes, while spleen sections showed germinal centre alterations consistent with B‐cell depletion, a characteristic feature of anti‐CD20 therapy (Figure S10B). Additionally, TRAP treatment did not significantly affect spleen weight (Figure S10C). Collectively, these findings indicate that TRAP demonstrates a favourable safety profile at the administered dose, with changes aligning with clinically acceptable effects of CD20‐targeted immunotherapy.

To further evaluate the therapeutic effect on mouse survival, Kaplan–Meier survival analysis was performed. MC38 tumour‐bearing mice were randomly assigned to three treatment groups (PBS, rAAV‐MCS + mCD20 Ab, and rAAV‐mCD20 + mCD20 Ab). Mice received intravenous injections of rAAV‐MCS or rAAV‐mCD20 every day for a total of two doses, followed 48 h later by administration of mCD20 Ab via the lateral tail vein. To enhance therapeutic efficacy, mCD20 Ab was subsequently administered intravenously once per week for two additional doses (Figure 6F). As a result, mice treated with rAAV‐mCD20 in combination with mCD20 Ab exhibited significantly prolonged survival compared with all control groups (Figure 6G).

The antitumour efficacy of TRAP was also evaluated in the microsatellite‐stable (MSS) syngeneic CT26 colorectal cancer model. Compared with the rAAV‐MCS + mCD20 Ab group, rAAV‐mCD20 + mCD20 Ab significantly inhibited CT26 tumour growth without affecting body weight (Figure S11A–C). Consistently, tumour volume and tumour weight at the endpoint were significantly reduced following TRAP treatment (Figure S11D,E). These findings demonstrate that TRAP retains antitumour activity in an MSS colorectal cancer model.

**FIGURE 7:**
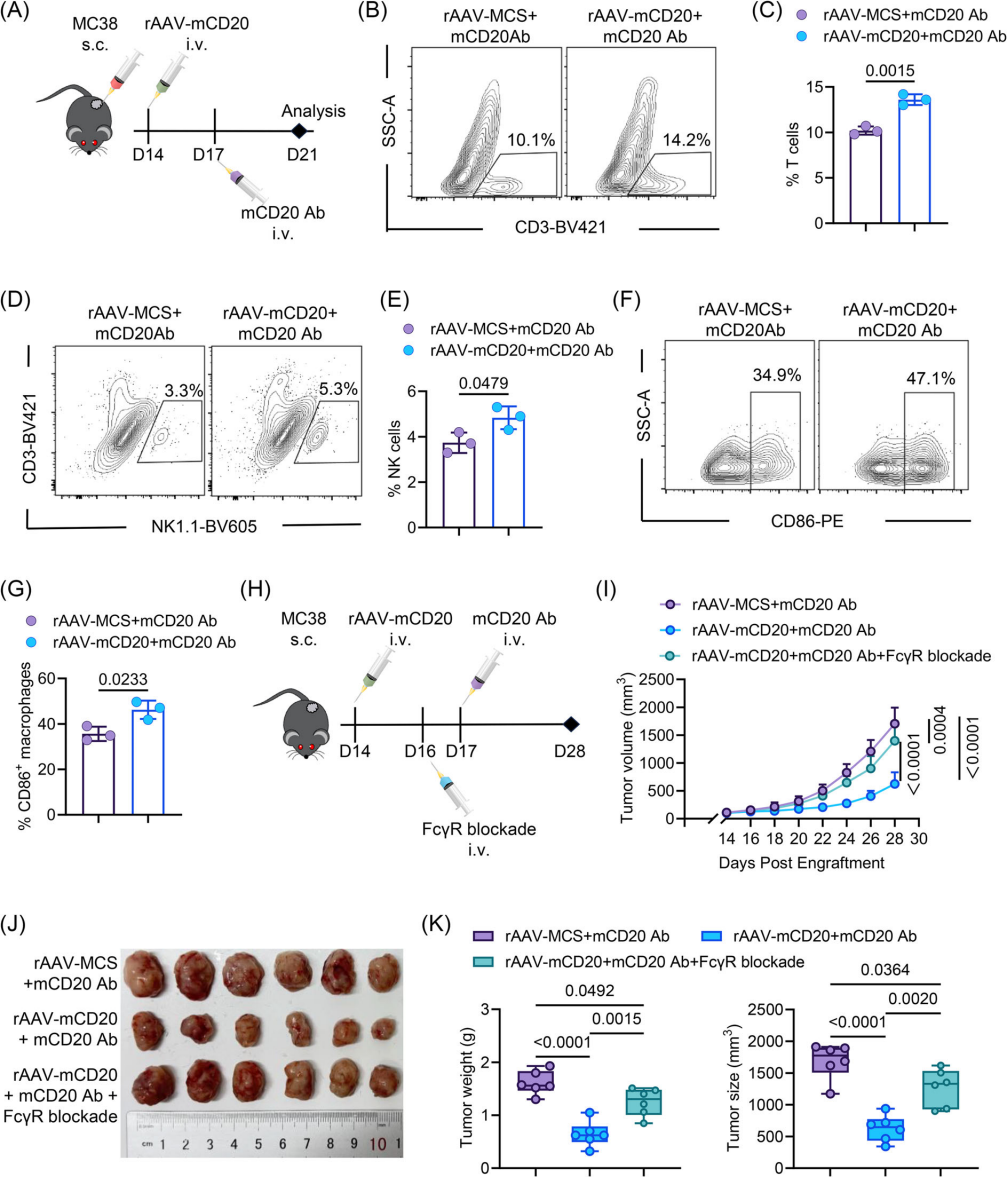
Effects of TRAP treatment on the tumour immune microenvironment in the immunocompetent MC38 model. (A) Schematic of the experimental schedule for tumour immune profiling. MC38 tumour‐bearing C57BL/6 mice were treated with rAAV‐MCS or rAAV‐mCD20 followed by anti‐mouse CD20 monoclonal antibody (mCD20 Ab), and tumours were collected 4 days after antibody administration for flow cytometric analysis. (B, C) Representative flow cytometry plots (B) and quantification (C) of CD3^+^ T cells as a percentage of viable CD45^+^ cells. (D, E) Representative flow cytometry plots (D) and quantification (E) of CD3^−^NK1.1^+^ NK cells as a percentage of viable CD45^+^ cells. (F, G) Representative flow cytometry plots (F) and quantification (G) of CD86^+^ macrophages. Macrophages were defined as CD45^+^CD11b^+^F4/80^+^ cells, and CD86^+^ macrophages are shown as a percentage of the macrophage population. Data in (C, E, G) are presented as mean ± SD (*n* = 3 per group). (H) Schematic of the FcγR blockade experiment. (I) Tumour growth curves of mice treated with rAAV‐MCS + mCD20 Ab, rAAV‐mCD20 + mCD20 Ab, or rAAV‐mCD20 + mCD20 Ab + FcγR blockade. (J) Images of excised tumours at the experimental endpoint. (K) Tumour weight and tumour volume at the experimental endpoint. Statistical analyses were performed as described in Section 2.20.

To further investigate the effects of TRAP treatment on the tumour immune microenvironment, we performed flow cytometric profiling of MC38 tumours 4 days after mCD20 Ab administration (Figure 7A). Compared with the rAAV‐MCS + mCD20 Ab group, treatment with rAAV‐mCD20 + mCD20 Ab significantly increased the proportion of CD3^+^ T cells among viable CD45^+^ cells (Figure 7B,C) and CD3^−^NK1.1^+^ NK cells among viable CD45^+^ cells (Figure 7D,E). The proportion of CD86^+^ cells within the CD45^+^CD11b^+^F4/80^+^ macrophage population was also significantly increased following TRAP treatment (Figure 7F,G), with the macrophage gating strategy shown in Figure S12A. In contrast, no significant differences were observed in the proportions of PMN‐MDSCs or M‐MDSCs within viable CD45^+^CD11b^+^ myeloid cells between the two groups (Figure S12B–D). These findings indicate that TRAP treatment is associated with enhanced T‐ and NK‐cell infiltration and a higher proportion of CD86^+^ macrophages in the tumour microenvironment.

To further evaluate the contribution of FcγR‐dependent effector mechanisms to the antitumour activity of TRAP, we performed an FcγR blockade experiment in the immunocompetent MC38 model (Figure 7H). FcγR blockade markedly attenuated the tumour growth inhibition induced by rAAV‐mCD20 + mCD20 Ab (Figure 7I). Consistently, mice receiving FcγR blockade exhibited significantly greater tumour weight and tumour volume at the experimental endpoint than those treated with rAAV‐mCD20 + mCD20 Ab alone (Figure 7J,K). These results support an important contribution of FcγR‐dependent immune effector mechanisms to TRAP‐mediated antitumour activity.

## 4 DISCUSSION

In this study, we present a novel therapeutic strategy termed tumour‐selective antigen delivery for repurposing antibody therapy (TRAP), which introduces a clinically validated target into tumour cells to potentially expand the use of an FDA‐approved monoclonal antibody for previously untargetable solid tumours. Using CD20 as a proof of concept, we demonstrated that the TRAP system enables selective expression of a membrane‐bound antigen on cancer cells in vitro, driven by our previously developed NF‐κB‐specific promoter (DMP).^20^, ^21^, ^22^, ^23^, ^31^ Consequently, the combination of DMP‐CD20 and rituximab significantly enhanced NK cell activation and induced ADCC across three different tumour cell lines. Among these, triple‐negative breast cancer (TNBC) was of particular interest due to its lack of estrogen and progesterone receptors as well as HER2 expression—features that render it unresponsive to existing targeted therapies and underscore the urgent need for novel treatment approaches.^32^ In addition to NK cell‐mediated ADCC, rituximab also promoted the phagocytosis of pAAV‐hCD20‐transfected HCT116 cells by PMA‐differentiated THP‐1 macrophage‐like cells in vitro. Given that macrophages represent one of the most abundant immune cell populations in the tumour microenvironment,^33^ this result suggests that TRAP‐induced CD20 expression may engage macrophage‐mediated Fc‐dependent antitumour activity. Moreover, TRAP‐mediated CD20 expression enabled antibody‐dependent activation of the complement system, as evidenced by C3 fragment deposition on tumour cells following OFA treatment in the presence of NHS. However, CDC was not uniformly observed across tumour cell lines, which we hypothesize may be attributable to the ability of certain solid tumours, including pancreatic cancer and triple‐negative breast cancer, to evade complement‐mediated lysis through the expression of membrane‐bound complement regulatory proteins (mCRPs).^34^, ^35^, ^36^ This requires further investigation and confirmation.

Moreover, TRAP showed consistent antitumour activity in HCT116 tumour spheroids, colorectal cancer PDOs, and in vivo tumour models, indicating that its therapeutic effect was maintained across increasingly complex experimental systems. The pronounced apoptotic response observed in PDOs is particularly relevant because patient‐derived organoids can preserve key molecular and phenotypic features of the original tumours and have been shown to recapitulate clinical treatment responses.^37^ Together, the concordant efficacy across these complementary models strengthens the proof‐of‐concept for TRAP and supports its potential applicability beyond conventional two‐dimensional tumour cell systems.

Flow cytometric profiling of MC38 tumours revealed enhanced infiltration of CD3^+^ T cells and NK cells, accompanied by a higher proportion of CD86^+^ macrophages following TRAP treatment. These findings suggest that TRAP may influence the tumour immune environment beyond direct antibody‐mediated tumour‐cell killing. Consistently, FcγR blockade attenuated the antitumour efficacy of TRAP, supporting an important contribution of FcγR‐dependent effector mechanisms, in line with established mechanisms of anti‐CD20 antibody therapy.^38^, ^39^ These immune changes may reflect broader immune engagement, although whether TRAP promotes tumour‐specific adaptive immunity or epitope spreading requires further investigation. However, because the present study was limited to three tumour cell lines, colorectal cancer PDOs, and three mouse models, including the MSS CT26 model, these findings should be considered proof‐of‐concept evidence, and the broader applicability of TRAP across solid tumour types requires further validation.

Although we performed these proof‐of‐concept studies using CD20 as a target, this strategy is adaptable to other antigens, including non‐human species‐specific constructs,^6^, ^7^, ^8^ which may further enhance antitumour activity by eliciting host immune responses against foreign proteins. Nevertheless, monoclonal antibodies targeting CD20, including rituximab, obinutuzumab, and ofatumumab, are clinically validated therapies with well‐characterized safety profiles.^40^, ^41^ Although systemic rituximab may deplete normal CD20‐positive B cells and impair de novo humoral responses, this on‐target effect is generally clinically manageable through vaccination before treatment, monitoring of B‐cell counts, serum immunoglobulin levels, and infections, and immunoglobulin replacement when clinically indicated.^42^, ^43^, ^44^ This established clinical experience supports the further development of CD20‐based TRAP.

In this study, we employed adeno‐associated virus (AAV) for in vivo antigen delivery, a strategy that has previously demonstrated considerable versatility.^14^, ^15^, ^16^, ^45^ The expression level of the introduced antigen could be modulated by adjusting the viral dose, thereby addressing the suboptimal efficacy of monoclonal antibodies resulting from low tumour antigen density. While certain small‐molecule inducers—such as Aurora kinase inhibitors, FOXO1 inhibitors, and chromatin modulators—can enhance CD20 expression, their systemic activity poses a considerable risk of off‐target toxicity.^46^, ^47^ Notably, we used a dose 100‐fold lower (10^1^ ^2^/kg body weight) than that typically administered in gene therapy for human genetic disorders (10^14^–10^1^ ^5^/kg body weight).^48^ Furthermore, rAAV‐MCS and rAAV‐mCD20 were administered at equivalent doses in mice and did not induce significant changes in key safety parameters, including body weight and markers of hepatic and renal function, thereby supporting the biosafety of this AAV‐based antigen delivery strategy for future clinical application.

A key concern in antigen‐targeted therapies is that, under sustained pressure from monoclonal antibody treatment, tumour cells may downregulate or lose antigen expression via various mechanisms. These include trogocytosis,^49^ an FcγR‐mediated process in which monocytes, macrophages, or neutrophils actively strip antibody–antigen complexes from the tumour cell surface,^50^, ^51^ as well as antigen internalization,^52^ and mutation‐driven loss.^53^ Notably, CD20 trogocytosis has been extensively documented in clinical and preclinical studies of rituximab and ofatumumab treatment, leading to rapid and pronounced loss of target antigen.^54^, ^55^ In such cases, repeated administration of viral vectors can restore antigen expression and potentially mitigate acquired resistance. However, AAV‐based gene therapies are limited by pre‐existing or treatment‐induced anti‐AAV antibodies, which may reduce vector efficacy. This issue can be partially controlled by B cell‐depleting agents, such as CD20‐targeting monoclonal antibodies, which may lower anti‐viral antibodies and thereby enhance the persistence and activity of this therapeutic combination.^56^, ^57^, ^58^ Moreover, several FDA‐approved agents—such as antibodies targeting the neonatal Fc receptor (FcRn), IgG‐cleaving endopeptidase, and IgG‐degrading enzymes—have shown efficacy in reducing or eliminating anti‐AAV antibodies, including neutralizing antibodies (NAbs),^59^, ^60^, ^61^, ^62^, ^63^ thereby supporting repeated antigen delivery in combination immunotherapy.

Beyond humoral immunity‐mediated constraints, incomplete AAV transduction represents an important consideration for antigen‐redirection strategies in solid tumours, as delivery barriers and tumour‐cell heterogeneity can limit uniform viral penetration and infection.^64^ Consistent with this limitation, our immunohistochemical and flow cytometric analyses showed heterogeneous CD20 expression and incomplete tumour‐cell coverage in vivo. As a result, antigen expression following AAV‐mediated delivery may be heterogeneous within tumours, potentially allowing antigen‐negative tumour cells to escape antibody‐mediated targeting. However, the present study did not directly assess long‐term antigen‐negative escape or tumour relapse, which requires further investigation. To address this limitation, the modular design of the TRAP platform enables rational combination strategies. For example, TRAP‐mediated antigen redirection could be combined with antibody–drug conjugates (ADCs) exhibiting bystander‐killing activity, thereby enabling elimination of neighbouring untransduced or antigen‐negative tumour cells through diffusion of cytotoxic payloads from antigen‐positive cells.^65^, ^66^ In addition, future optimization strategies, including AAV capsid engineering to improve tumour transduction efficiency, may further enhance therapeutic durability and translational potential in heterogeneous solid tumours.^67^ Given that the current PBMC–tumour co‐implantation model provides only short‐term proof‐of‐concept evidence, future studies using CD34^+^ hematopoietic stem cell‐humanized mouse models will be needed to evaluate immune‐cell trafficking, tumour infiltration, and the long‐term systemic efficacy of TRAP combined with rituximab.

Considering the diversity of immunotherapeutic strategies, this cooperative approach is not confined to CD20 and monoclonal antibodies. More broadly, our study addresses the challenge of antigen heterogeneity and scarcity in solid tumours and establishes a tumour‐selective, clinically validated target‐modification platform. This in situ tumour‐target engineering strategy may also be integrated with next‐generation immunotherapies such as BsAbs,^68^, ^69^, ^70^, ^71^ and CAR‐T cells,^41^ highlighting the potential of rAAV as a versatile framework for combinatorial cancer treatment.

## 5 CONCLUSIONS

Taken together, this study introduces TRAP, a tumour‐targeting strategy that integrates rAAV‐mediated antigen delivery with clinically validated monoclonal antibody therapy to overcome the limitations of target availability in solid tumours. By enabling tumour‐selective antigen expression, TRAP broadens the applicability of approved antibody therapies and elicits effective antitumour responses across multiple experimental models, including both in vitro cell cultures, three‐dimensional spheroids, and patient‐derived organoids, as well as in vivo tumour models. These findings provide proof‐of‐concept evidence for the TRAP strategy in the tested tumour models. Further validation in additional tumour types and clinically relevant models is required to determine its broader applicability and translational potential.

## AUTHOR CONTRIBUTIONS

Z.K. under the direction of J.W., designed and performed experiments, analyzed the data, and wrote the first version of the manuscript. Y.W., Y.Z., and L.W performed part of the experiments. J.W., F.Z., and Y.S. designed experiments and supervised the study. Z.K. and J.W. wrote the manuscript. All authors contributed to the critical revision of the manuscript and approved the final version.

## CONFLICT OF INTEREST STATEMENT

The authors declare no conflict of interest.

## ETHICS STATEMENT AND CONSENT TO PARTICIPATE

All animal experiments were performed under protocols approved by the Animal Experimentation and Ethics Committee of Southeast University. All tissues were obtained from patients who had undergone surgery at the Nanjing Hospital of Chinese Medicine Affiliated to Nanjing University of Chinese Medicine, with the consent of the patients and approval of the Nanjing Hospital of Chinese Medicine Committee for Ethical Review of Research involving Human Subjects (approval number: KY2025052). All the patients signed an informed consent form.

## CONSENT FOR PUBLICATION

The authors have nothing to report.

## Supporting information



Supplementary Information

Supplementary Information

## Data Availability

The authors declare that the main data supporting the findings of this study are available within the Article and the Supporting Information or available from the corresponding author upon reasonable request. The source data underlying Figures 1b–d, 2b,d,e, 3b,d,e, 4c,d,f, 5b,c,e,f,h, 6b,c,e,g, and 7c,e,g,i,k and Figures S3b, S5a,b, S7b, S8a,b,c,f, S9b,c, S10a,c, S11b,c,e, and S12c,d are provided with the paper as a Source Data file.
