# Supplementary material for: Cancer cell‐selective ectopic expression of CD20 as an antigen enables rituximab repurposing for solid tumour immunotherapy
Source: Clin Transl Med. 2026 Aug 31;16(9):e70804. doi: 10.1002/ctm2.70804 (PMC13528618; doi:10.1002/ctm2.70804)
Supplement: Supplementary file 1 [file CTM2-16-e70804-s001.pdf]

Supplementary Information

Cancer cell-selective ectopic expression of CD20 as an antigen enables rituximab repurposing for solid tumor immunotherapy

Ziyan Kong[1], Yile Wang[1], Yunqi Zhao[1], Lu Wang[2], Zhimin Fan[2], Yongqian Shu[3], Jinke Wang[1*]

[1] *School of Biological Science and Medical Engineering, Southeast University, Nanjing 210096, China*

[2] *Jiangsu Clinical Innovation Center for Anorectal Diseases of T.C.M, Nanjing Hospital of Chinese Medicine Affiliated to Nanjing University of Chinese Medicine, Nanjing 210022, China*

[3] *Department of Oncology, The First Affiliated Hospital of Nanjing Medical University, Nanjing 210029, China*

Supplementary Table 1 Primer sequences used for plasmid construction

Name	Primer Sequence (5'-3')
Mouse CD20-F	GAATTCGCCACCATGAGTGG
Mouse CD20-R	GGATCCTTAAGGAGCGATCTCA
Human CD20-F	AGCTCGACTTCCAGGAATTCACTAGCACAACCCCAGA
Human CD20-R	CGACTCTAGAGGACTCCTTAAGGAGAGGCTGCTATTTTCT
WPRE-F	GAGTCGACCTGCAGAAGCTTAACTCAACCTCTGGATT
WPRE-R	TGCTCGAGGCAAGCTCGCGGGAGGCGGGCCCAAAG

Supplementary Table 2 Primer sequences used for qPCR

Name	Primer Sequence (5'-3')
AAV-F	TGCATGACCAGCTTCAAGCTA
AAV-R	GAACAGGGAGAGGAGCAGATG
Mouse RELA-F	TGCATTCCTCCACTTAAACGC
Mouse RELA-R	ACAATCTCTGTCTGTAGGCGC
Mouse GAPDH-F	TCACCATCTTCCAGGAGCGC
Mouse GAPDH-R	CTGCTCCTGGAAGATGGTGA
Human RELA-F	CCTGGAGCAAGGACTCAGCA
Human RELA-R	ATGCACAGCAGGACAATGGG
Human GAPDH-F	ATTTTGGAGGGATCTCGCTCC
Human GAPDH-R	CTCCCTCTTCCGTTCCAGTTT
Mouse CD20-F	CTTTCCCGAGGACGGCCTAC
Mouse CD20-R	ATGGCAGTGGAGTCAGGAAT
Human CD20-F	CTTTGGGGCGGTCCCAGATT
Human CD20-R	AGATTTGGGGTGCTGAGCAG

Supplementary Table 3 Flow cytometry staining panel

Marker	Tag	Manufacturer	Clone
Live dead staining			
7-AAD	7-AAD	BioLegend	—
Zombie Aqua Fixable Viability	Zombie Aqua	BioLegend	—
Surface staining (Human)			
Human TruStain FcX	—	BioLegend	—
Human CD20	APC	BioLegend	2H7
Human CD45	FITC	BioLegend	HI30
Human CD3	PE	BioLegend	SK7
Human CD56	APC	BioLegend	5.1H11
Human CD107a	FITC	BioLegend	H4A3
Human C3b/iC3b	FITC	BioLegend	3E7
Surface staining (Mouse)			
Mouse CD16/32	—	BioLegend	S17011E
Mouse CD20	PE	BioLegend	QA18A73
Mouse CD45	FITC	BioLegend	30-F11
Mouse CD3	Brilliant Violet 421	BioLegend	17A2
Mouse NK1.1	Brilliant Violet 605	BioLegend	PK136
Mouse CD11b	PerCP/Cyanine5.5	BioLegend	M1/70
Mouse F4/80	Alexa Fluor 700	BioLegend	BM8
Mouse CD86	PE	BioLegend	A17199A
Mouse Ly-6G	Brilliant Violet 650	BioLegend	1A8
Mouse Ly-6C	APC	BioLegend	HK1.4
Mouse CD31	APC	BioLegend	MEC13.3
Intracellular staining†			
IFN-γ	FITC	BioLegend	4S. B3

† Cells were fixed and permeabilized using Cyto-Fast™ Fix/Perm Buffer Set (BioLegend)

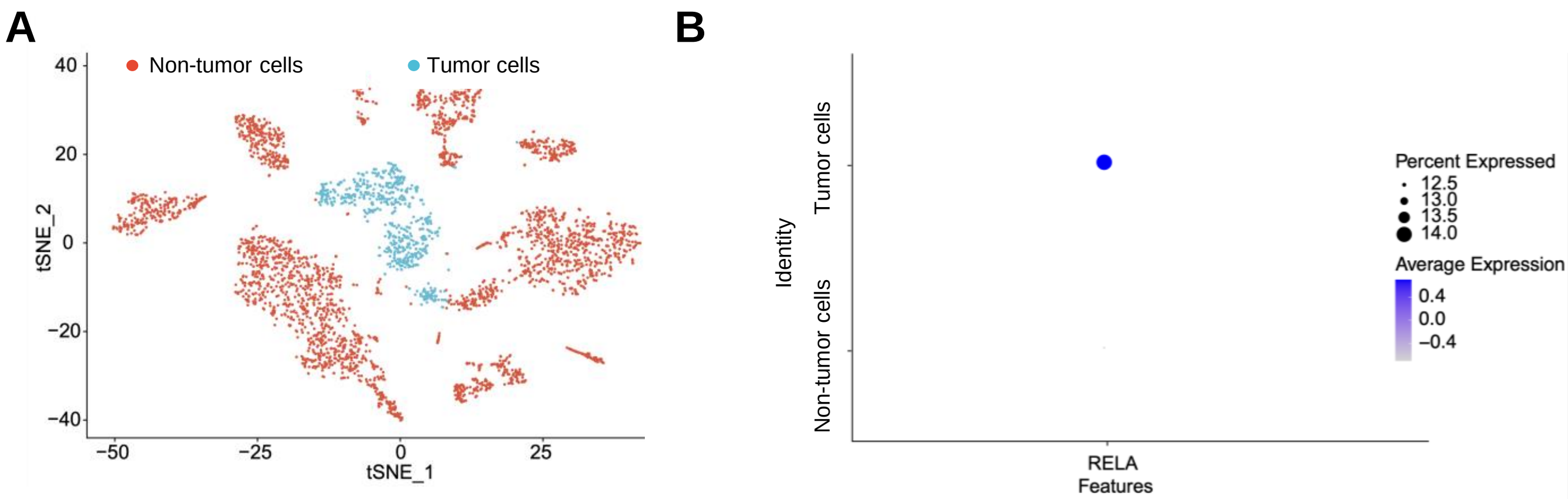

Supplementary Figure 1. Single-cell RNA-sequencing analysis of RELA expression in pancreatic ductal adenocarcinoma. (A) t-SNE visualization of tumor and non-tumor cells from the publicly available single-cell RNA-sequencing dataset GSE335452. (B) Dot plot showing RELA expression in tumor and non-tumor cells. Dot size represents the percentage of cells expressing RELA, and color intensity represents the average expression level.

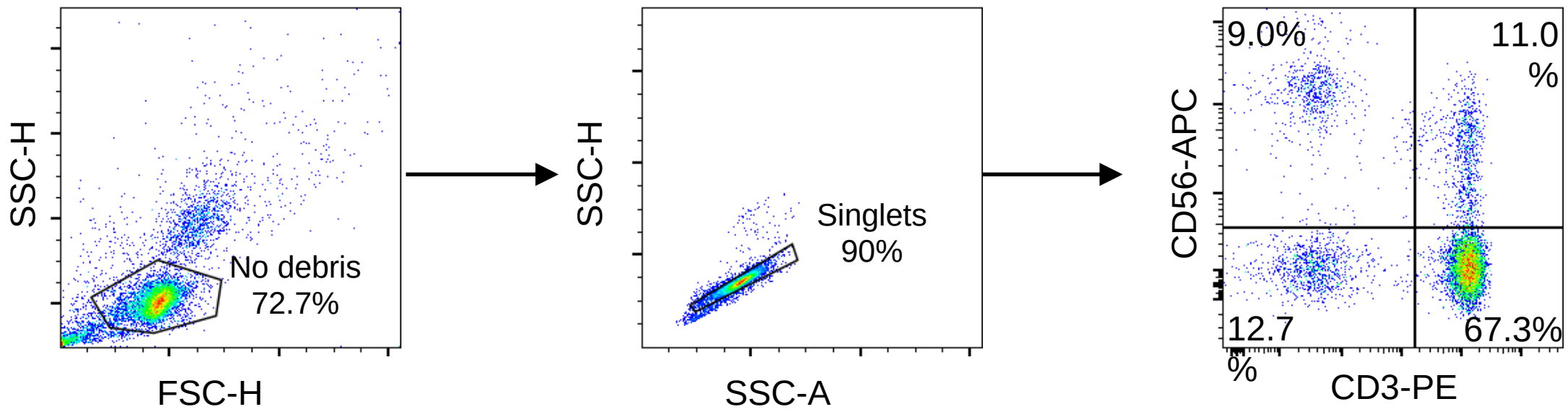

Supplementary Figure 2. Flow-cytometry gating strategy for NK-cell analysis. Peripheral blood mononuclear cells (PBMCs) were first gated on forward scatter (FSC-H) versus side scatter (SSC-H) to exclude debris, followed by singlet discrimination using SSC-A versus SSC-H. Within the singlet population, NK cells were identified as $CD3^{-}CD56^{+}$ using anti-CD3-PE and anti-CD56-APC staining. This gating strategy was applied throughout the NK-cell degranulation (CD107a) and IFN-γ assays presented in Figure 2. Representative plots show the sequential gating steps, with the final NK-cell population highlighted in the CD3/CD56 quadrant.

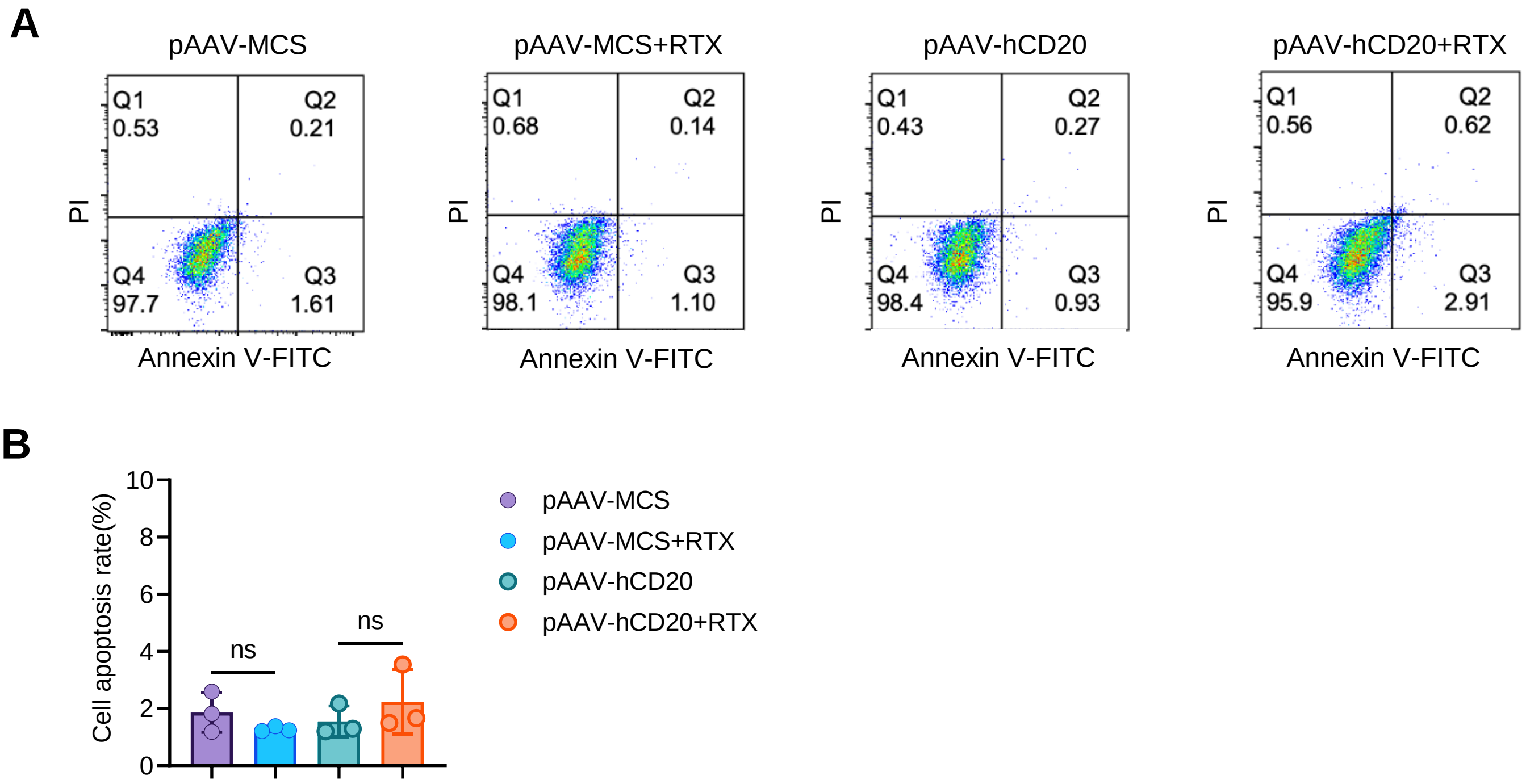

Supplementary Figure 3. Assessment of direct rituximab-induced apoptosis in CD20-expressing HCT116 cells. (A) Representative Annexin V-FITC/PI flow cytometry plots of HCT116 cells transfected with pAAV-MCS or pAAV-hCD20 and treated with or without rituximab (RTX, 10 μg/mL) for 4 h in the absence of PBMCs. (B) Quantification of apoptotic cells, defined as Annexin V^{+} cells, including Annexin V^{+}PI^{-} and Annexin V^{+}PI^{+} populations. Data are presented as mean ± SD from three independent experiments. Statistical significance was analyzed by one-way ANOVA with Tukey's multiple-comparison test; ns, not significant.

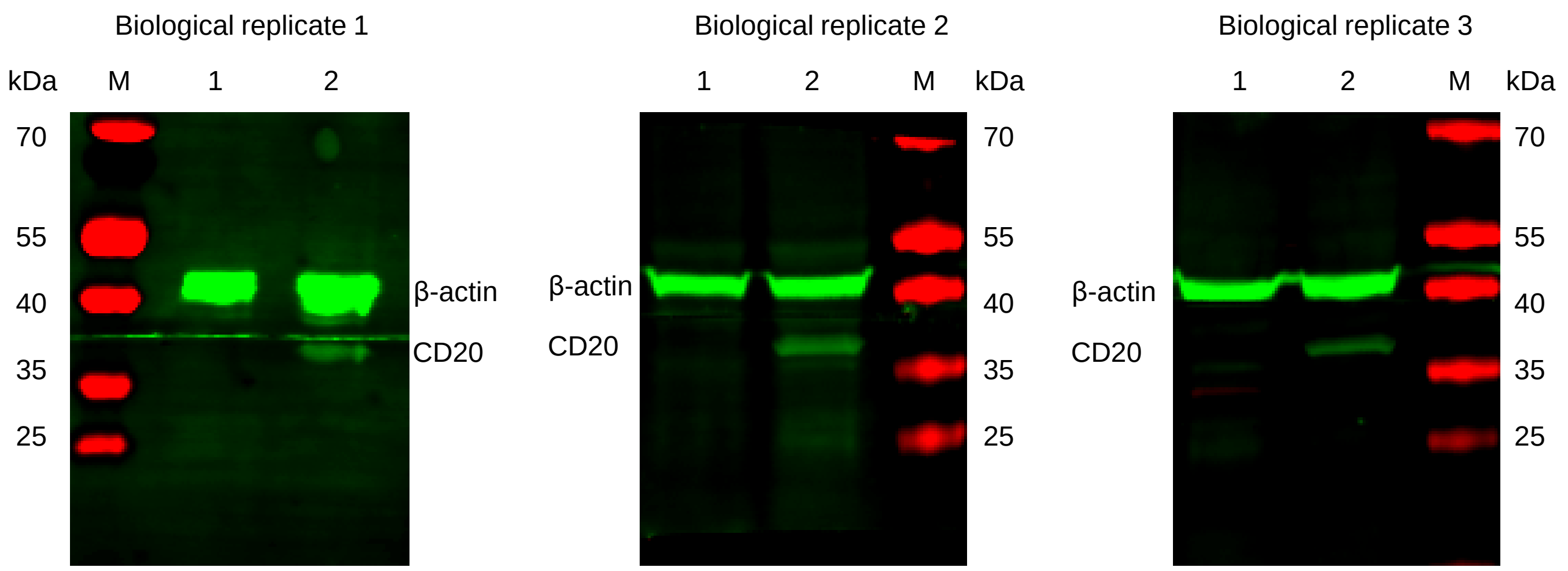

Supplementary Figure 4. CD20 expression in HCT116 tumor spheroids after rAAV transduction. Western blot analysis of CD20 expression in HCT116 spheroids 72 h after infection with rAAV-MCS or rAAV-hCD20. β-actin served as a loading control. The three panels show results from three independent biological replicates. M, protein molecular-weight marker; lane 1, rAAV-MCS; lane 2, rAAV-hCD20. Molecular weights are indicated in kilodaltons (kDa). CD20 and β-actin were detected at approximately 37 and 42 kDa, respectively.

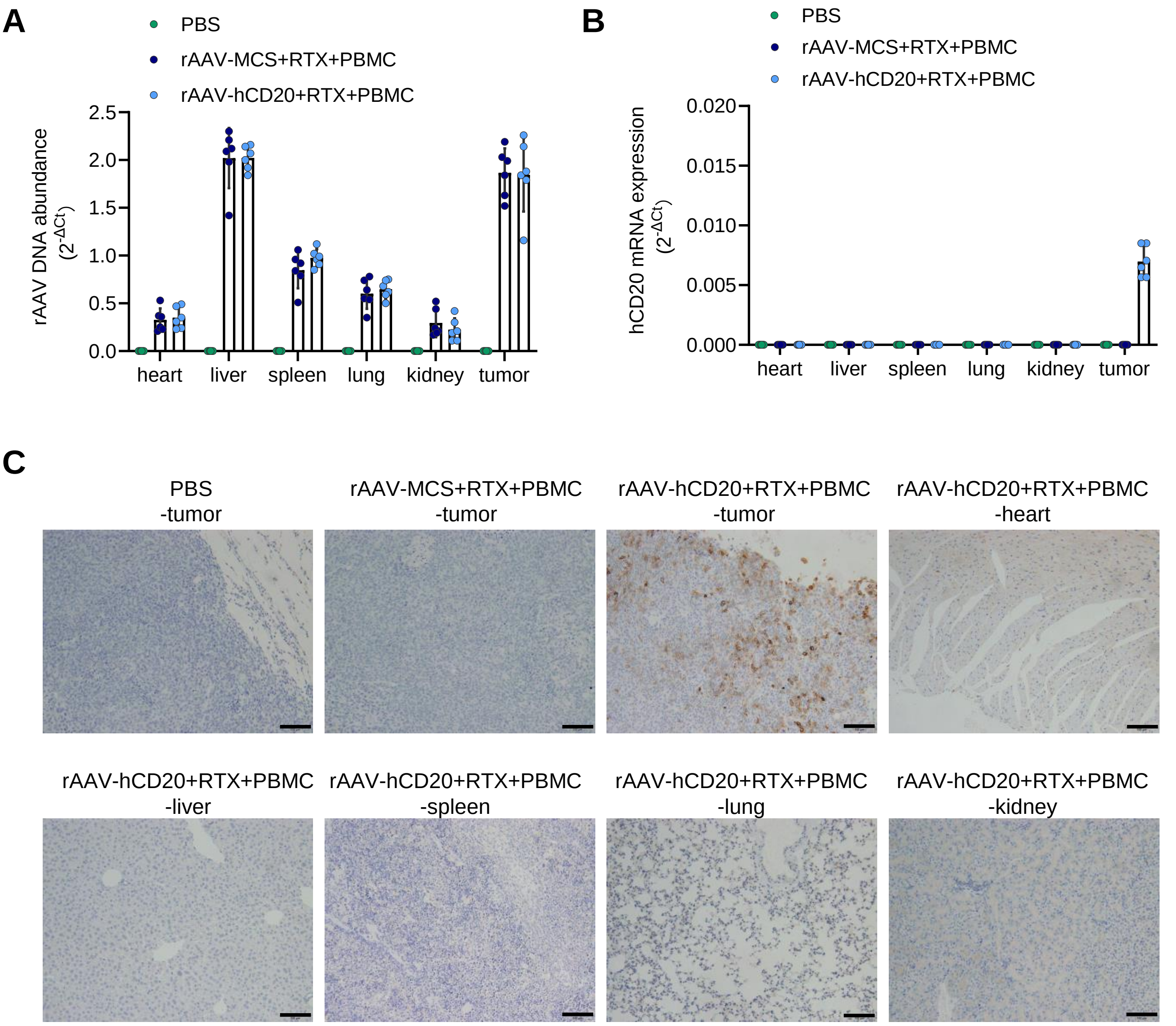

Supplementary Figure 5. Biodistribution and tumor-selective expression of rAAV-hCD20 in NCG mice

(A) Quantification of rAAV DNA in heart, liver, spleen, lung, kidney, and tumor tissue by qPCR (n = 6). (B) hCD20 mRNA expression in major organs and tumor tissue (n = 6). (C) Representative immunohistochemical staining of CD20 in tumors from the indicated treatment groups and in major organs from rAAV-hCD20 + RTX + PBMC-treated mice. Scale bars, 100 μm. Data are shown as mean ± SD.

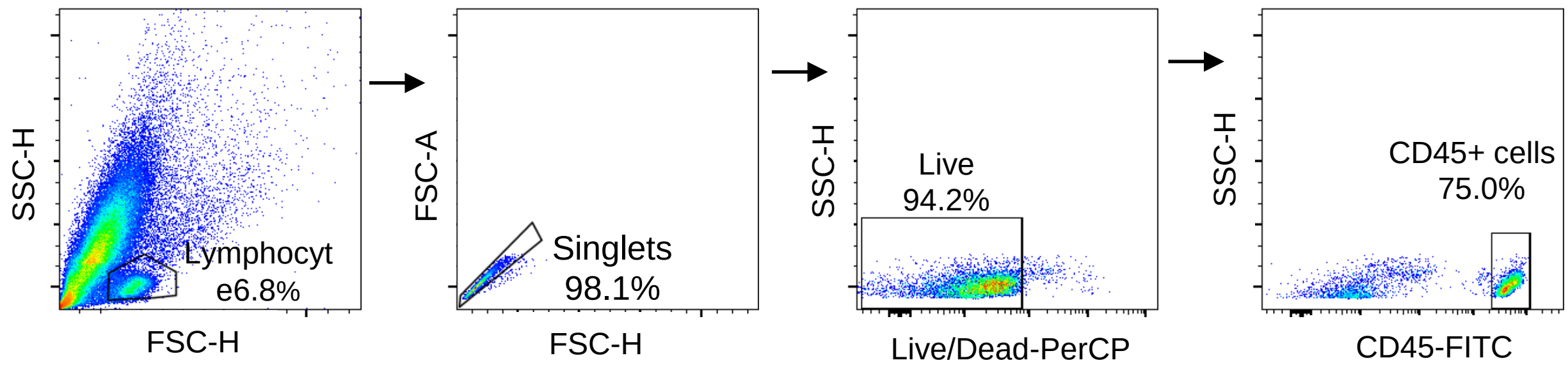

Supplementary Figure 6. Flow cytometry gating strategy for analysis of tumor-infiltrating lymphocytes. Sequential gating showing identification of live $CD45^+$ cells and subsequent determination of NK cells as $CD3^-CD56^+$ events. Representative plots are shown.

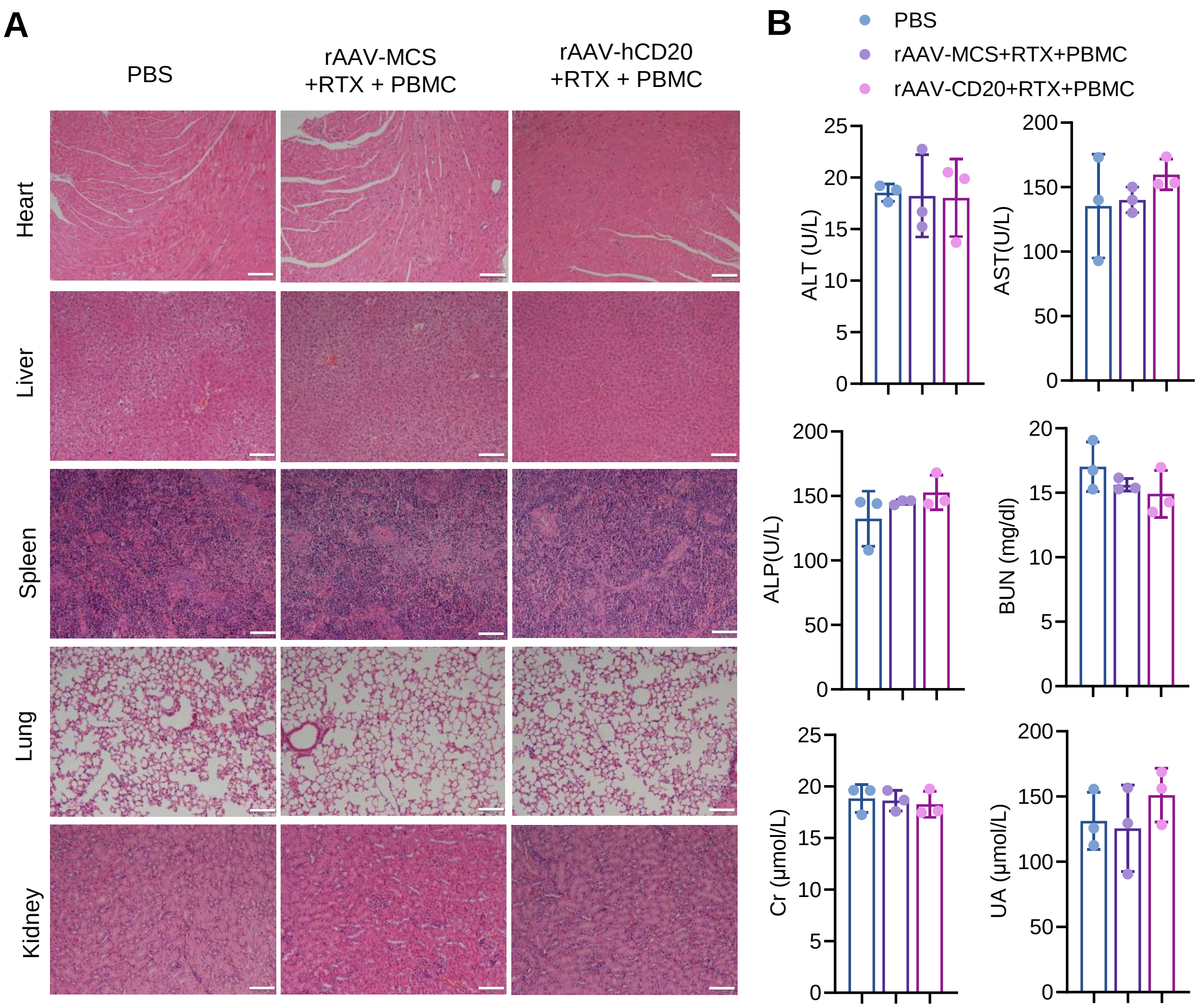

Supplementary Figure 7. Safety evaluation of TRAP in NCG mice. (A) Hematoxylin and eosin (H&E) staining of heart, liver, spleen, lung, and kidney tissues from each group. Scale bar, 100 μm. (B) Serum biochemistry analysis of liver function markers (ALT, AST, ALP) and kidney function markers (BUN, creatinine [Cr], uric acid [UA]) in mice treated with PBS, rAAV-MCS +RTX+ PBMCs, or rAAV-hCD20 + RTX + PBMCs (n = 3). Data are shown as mean ± SD.

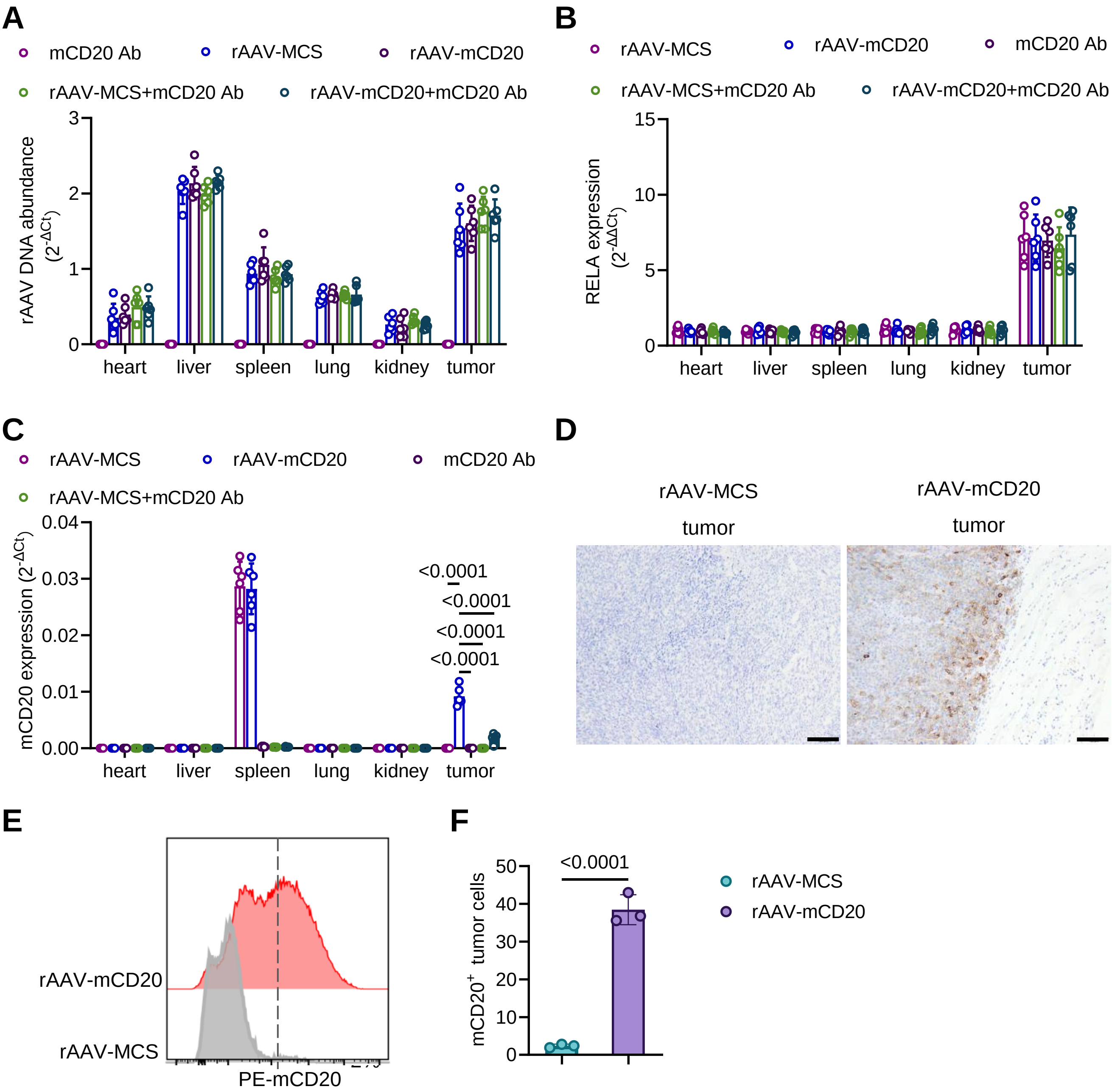

Supplementary Figure 8. Biodistribution and target gene expression in C57BL/6 mice bearing MC38 tumors. (A) rAAV DNA abundance in major organs and tumor tissue (n = 6). (B) RELA mRNA expression in heart, liver, spleen, lung, kidney, and tumor tissue (n = 6). (C) mCD20 mRNA expression in the indicated tissues (n = 6). (D) Representative immunohistochemical staining of mCD20 in MC38 tumor sections from mice treated with rAAV-MCS or rAAV-mCD20. Scale bars, 100 μm. (E) Representative flow cytometry histograms showing surface mCD20 expression within viable $CD45^-$ tumor cells from MC38 tumor-bearing C57BL/6 mice. When tumors reached approximately 100 mm^3, mice received a tail-vein injection of rAAV-MCS or rAAV-mCD20 (5×10^{10} vg/mouse), and tumors were collected one week later for flow cytometric analysis. (F) Quantification of $mCD20^+$ cells within the viable $CD45^-$ tumor-cell population in the rAAV-MCS and rAAV-mCD20 groups (n = 3 per group). Data are shown as mean ± SD. Statistical significance was analyzed by one-way ANOVA with Tukey's test in (C) and by an unpaired two-tailed Student's t-test in (F).

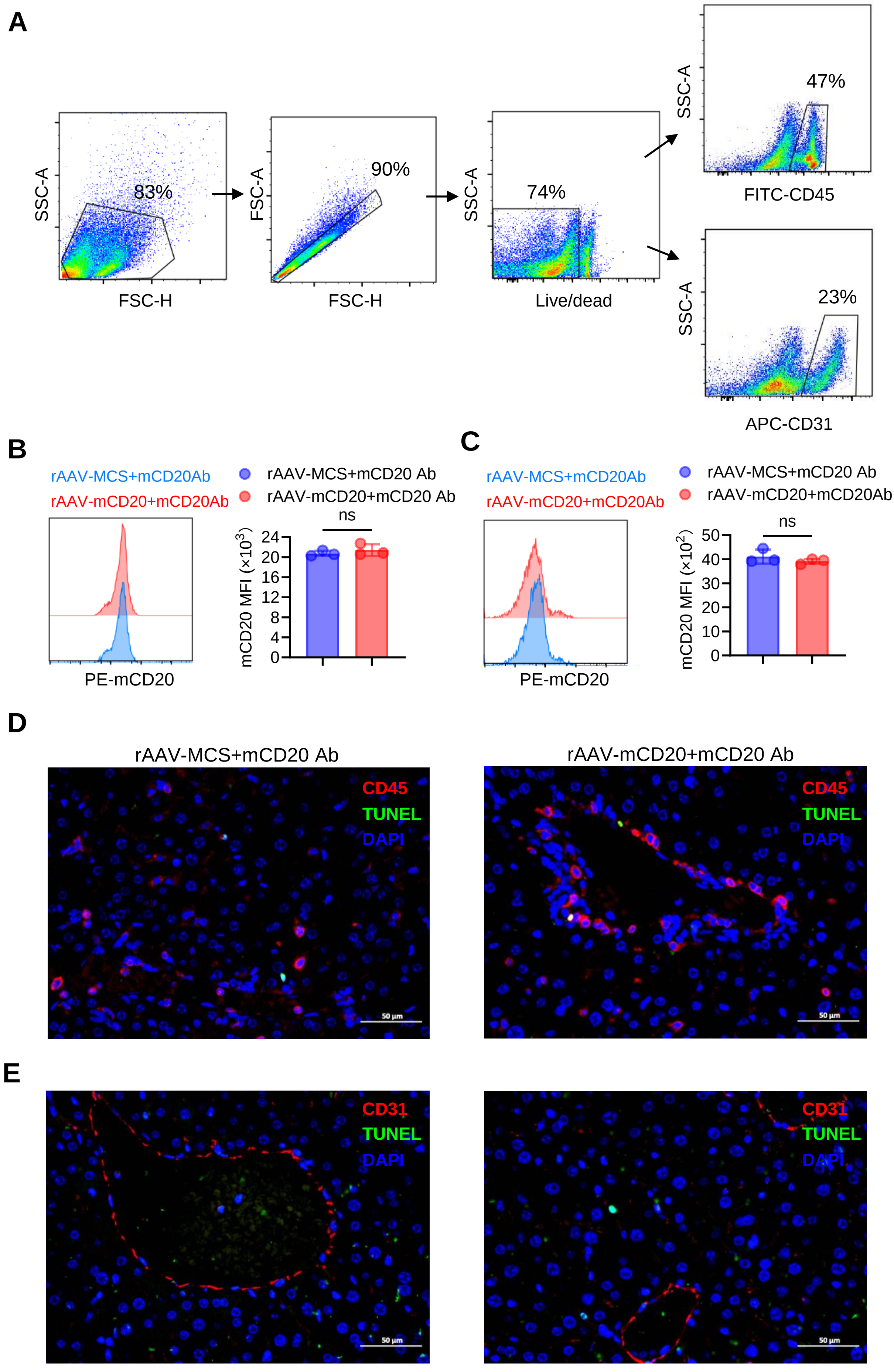
A
SSC-A
FSC-H
83%
FSC-A
FSC-H
90%
SSC-A
Live/dead
74%
SSC-A
FITC-CD45
47%
SSC-A
APC-CD31
23%
B
rAAV-MCS+mCD20Ab
rAAV-mCD20+mCD20Ab
PE-mCD20
rAAV-MCS+mCD20 Ab
rAAV-mCD20+mCD20 Ab
ns
mCD20 MFI (×10³)
C
rAAV-MCS+mCD20Ab
rAAV-mCD20+mCD20Ab
PE-mCD20
rAAV-MCS+mCD20 Ab
rAAV-mCD20+mCD20Ab
ns
mCD20 MFI (×10²)
D
rAAV-MCS+mCD20 Ab
rAAV-mCD20+mCD20 Ab
CD45
TUNEL
DAPI
E
CD31
TUNEL
DAPI

Supplementary Figure 9. Evaluation of mCD20 expression and apoptosis in hepatic immune and $CD31^+$ cell populations. (A) Flow cytometric gating strategy for analysis of hepatic $CD45^+$ and $CD31^+$ cell populations after exclusion of debris, doublets, and dead cells. **(B)** Representative flow cytometric histograms and quantification of surface mCD20 expression in hepatic $CD45^+$ immune cells from MC38 tumor-bearing mice treated with rAAV-MCS + mCD20 Ab or rAAV-mCD20 + mCD20 Ab. **(C)** Representative flow cytometric histograms and quantification of surface mCD20 expression in hepatic $CD31^+$ cells from the indicated groups. Data are presented as mean ± SD (n = 3 per group). Statistical significance was analyzed using an unpaired two-tailed Student's *t*-test; ns, not significant. **(D)** Representative immunofluorescence images of liver sections stained for CD45 (red), TUNEL (green), and DAPI (blue). **(E)** Representative immunofluorescence images of liver sections stained for CD31 (red), TUNEL (green), and DAPI (blue). Scale bars, 50 μm.

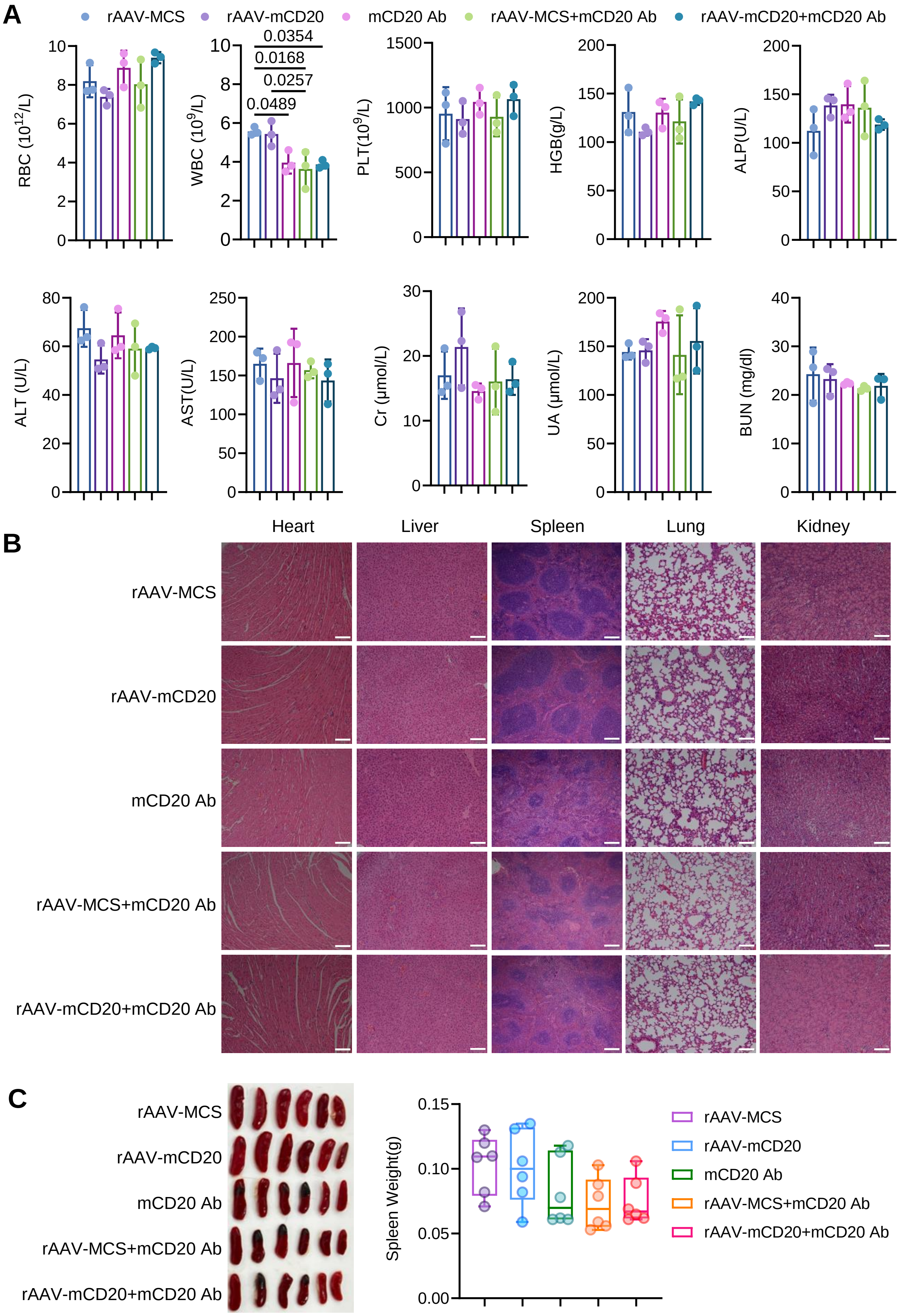
A
rAAV-MCS
rAAV-mCD20
mCD20 Ab
rAAV-MCS+mCD20 Ab
rAAV-mCD20+mCD20 Ab
RBC (10^{12}/L)
WBC (10^{9}/L)
0.0354
0.0168
0.0257
0.0489
PLT(10^{9}/L)
HGB(g/L)
ALP(U/L)
ALT (U/L)
AST(U/L)
Cr (μmol/L)
UA (μmol/L)
BUN (mg/dl)
B
Heart
Liver
Spleen
Lung
Kidney
rAAV-MCS
rAAV-mCD20
mCD20 Ab
rAAV-MCS+mCD20 Ab
rAAV-mCD20+mCD20 Ab
C
rAAV-MCS
rAAV-mCD20
mCD20 Ab
rAAV-MCS+mCD20 Ab
rAAV-mCD20+mCD20 Ab
Spleen Weight(g)
0.15
0.10
0.05
0.00
rAAV-MCS
rAAV-mCD20
mCD20 Ab
rAAV-MCS+mCD20 Ab
rAAV-mCD20+mCD20 Ab

Supplementary Figure 10. Hematological and histological safety evaluation of TRAP in C57BL/6 mice. (A) Hematological and serum biochemistry parameters including red blood cells (RBC), white blood cells (WBC), platelets (PLT), hemoglobin (HGB), ALT, AST, ALP, BUN, Cr, and UA (n = 6). (B) H&E staining of heart, liver, spleen, lung, and kidney tissues from each group. Scale bar, 100 μm. (C) Images and weight of spleens from mice treated with rAAV-MCS, rAAV-mCD20, mCD20 Ab, rAAV-MCS + mCD20 Ab, or rAAV-mCD20 + mCD20 Ab (n = 6). Data are shown as mean ± SD. Statistical significance was analyzed by one-way ANOVA with Tukey's test in (B).

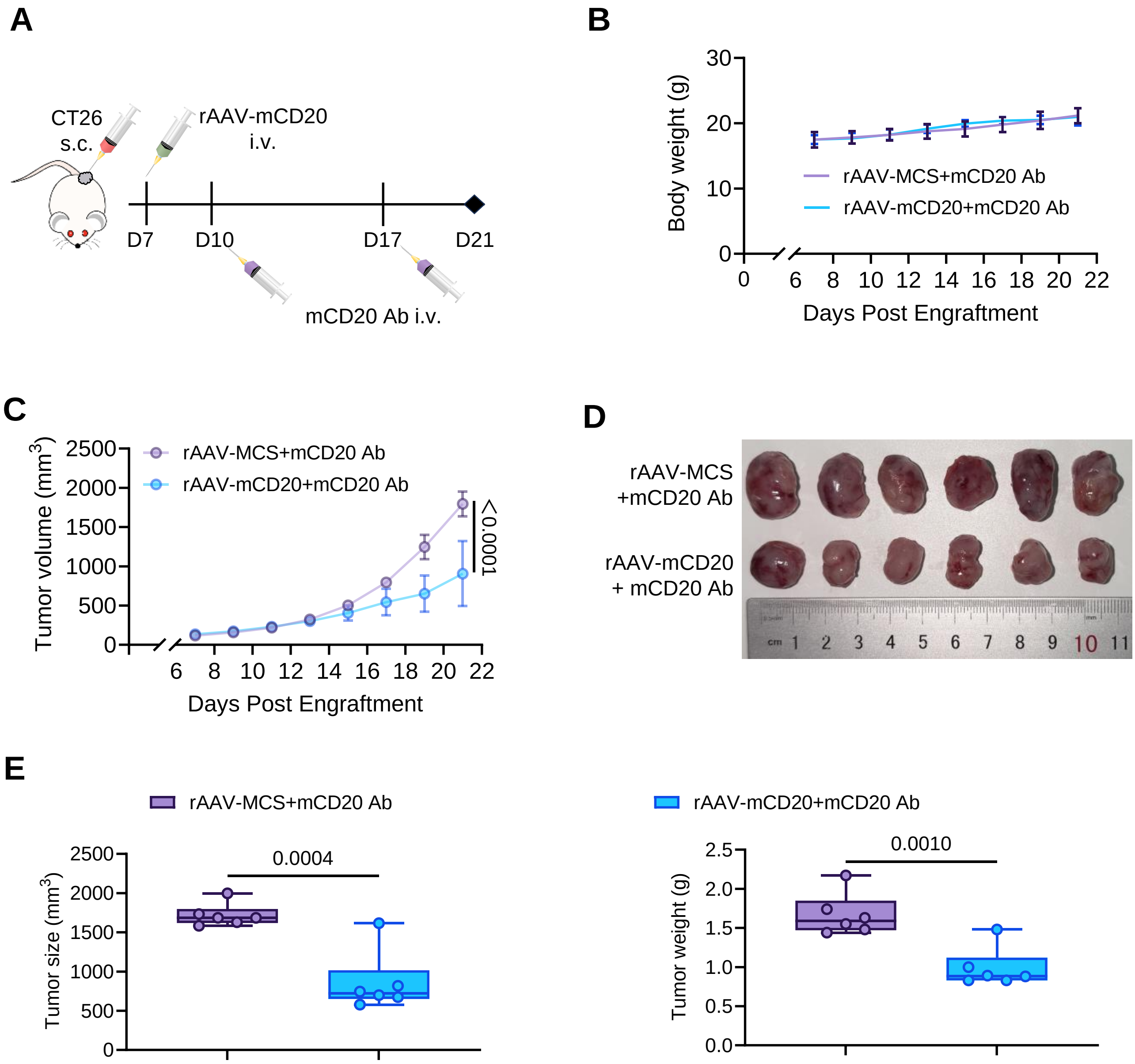

Supplementary Figure 11 Antitumor efficacy of TRAP in the MSS CT26 colorectal cancer model. (A) Schematic of the experimental design. CT26 tumor-bearing mice were treated with rAAV-MCS or rAAV-mCD20 followed by intravenous administration of anti-mouse CD20 monoclonal antibody (mCD20 Ab) as indicated. (B) Body weight of mice during treatment. (C) Tumor growth curves of mice treated with rAAV-MCS + mCD20 Ab or rAAV-mCD20 + mCD20 Ab. (D) Images of excised tumors collected from each group at the experimental endpoint. (E) Tumor volume (left) and tumor weight (right) at the experimental endpoint. Data in (B, C) are presented as mean ± SD (n = 6 per group). Statistical analyses were performed as described in the Methods.

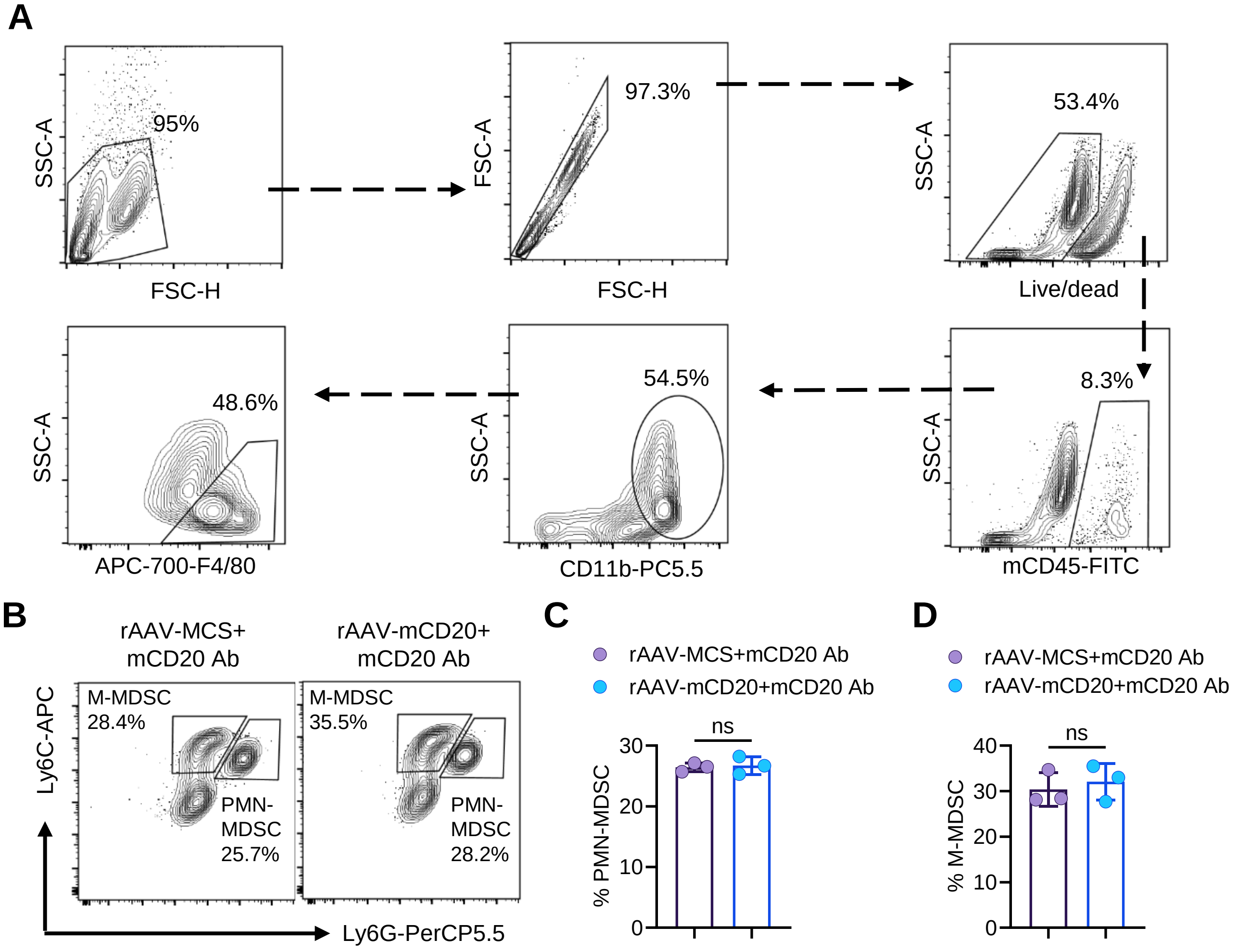

Supplementary Figure 12. Flow cytometric gating strategy and analysis of tumor-infiltrating myeloid cell populations in the MC38 model. (A) Representative flow cytometric gating strategy for tumor-infiltrating immune cells. Cells were sequentially gated to exclude debris, doublets, and dead cells, followed by identification of viable $CD45^+$ leukocytes and $CD11b^+$ myeloid cells. Macrophages were identified as $CD45^+CD11b^+F4/80^+$ cells. (B) Representative flow cytometry plots showing the identification of myeloid-derived suppressor cell (MDSC) subsets within viable $CD45^+CD11b^+$ cells. PMN-MDSCs were defined as $Ly6G^+$Ly6C^lo cells, whereas M-MDSCs were defined as $Ly6G^-$Ly6C^hi cells. (C, D) Quantification of tumor-infiltrating PMN-MDSCs (C) and M-MDSCs (D), expressed as percentages of viable $CD45^+CD11b^+$ myeloid cells. Data are presented as mean ± SD (n = 3 per group). Statistical significance was assessed using an unpaired two-tailed Student's t-test.
